# Unexpectedly Strong Electrostatic Screening in Nano‐Ion Solutions

**DOI:** 10.1002/anie.1870244

**Published:** 2026-08-20

**Authors:** Thomas Tilger, Esther Ohnesorge, Hayden Robertson, Michael Ludwig, Michalis Tsintsaris, Regine von Klitzing

**Affiliations:** ^1^ Soft Matter at Interfaces, Department of Physics Technical University of Darmstadt Darmstadt Germany

**Keywords:** colloidal probe atomic force microscopy, direct force measurements, electrostatic interactions, polyoxometalates, superchaotropes

## Abstract

Electrostatic interactions in soft matter and physical chemistry are fundamentally governed by the size and charge of the interacting species. The two polyoxometalates (POMs) with the Keggin structure phosphotungstate ${\left[{\mathrm{PW}}_{12}O_{40}\right]}^{3-}$ $\left({\mathrm{PW}}^{3-}\right)$ and silicotungstate ${\left[{\mathrm{SiW}}_{12}O_{40}\right]}^{4-}$ $\left({\mathrm{SiW}}^{4-}\right)$ occupy an intermediate position between classical ions and colloidal particles. Earlier attempts to categorize POMs within this spectrum have led to inconsistent results. Thus, herein we aim to shed light on the true nature of POMs based on their electrostatic screening behavior. Colloidal probe atomic force microscopy (CP‐AFM) is employed to measure the interaction forces between charged silica microspheres across various aqueous POM solutions. Initially, pure ${\mathrm{PW}}^{3-}$ and ${\mathrm{SiW}}^{4-}$ solutions are investigated over a wide range of concentrations. The CP‐AFM data reveal unexpectedly strong electrostatic screening by both POMs at millimolar concentrations, which we attribute to ion aggregation. These results are more consistent with an ionic nature of the POMs than with a colloidal character. Additional investigations of ${\mathrm{SiW}}^{4-}$ in the presence of NaCl as a background electrolyte support the original hypothesis and confirm that upon addition of salt, the same effect as for the pure POM solutions at higher concentrations is observed; the electrostatic screening is more effective than expected.

## Graphical abstract

Colloidal probe AFM measurements reveal unexpectedly strong electrostatic screening in aqueous solutions of the polyoxometalates phosphotungstate ${\left[{\mathrm{PW}}_{12}O_{40}\right]}^{3-}$ and silicotungstate ${\left[{\mathrm{SiW}}_{12}O_{40}\right]}^{4-}$. The screening is modulated by the polyoxometalate concentration and the presence of a background electrolyte, being more consistent with an ionic nature of the polyoxometalates than with a colloidal character.

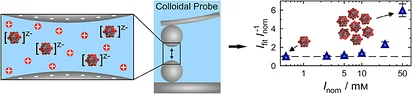

## 1 Introduction

Large, nanometer‐sized ions with only a few delocalized charges and high polarizability (often abbreviated as “nano‐ions”) have attracted significant attention over the last years. The two main classes of nano‐ions, polyoxometalates (POMs, such as Keggin ions) and ionic boron clusters (i.e., dodecaborates and metallacarboranes), share their strong affinity to various types of hydrated soft matter. They readily adsorb to non‐ionic oligo‐ [1] and polymers [2, 3, 4], including cross‐linked microgels [5]. Strong interactions were also found with non‐ionic short‐chain amphiphiles [6] and surfactants, in bulk [7, 8, 9, 10, 11, 12] and at interfaces [7, 13, 14]; they are able to stabilize foam films and foams even more efficient than classical ionic surfactants [13, 14]. In biological systems, nano‐ions adsorb to lipid membranes [15], strongly affecting their structure, and proteins [16]. The main driving force behind these phenomena was found to be the chaotropic effect, that is, the water‐mediated driving force for ion binding associated with the ability of ions to disrupt the hydrogen‐bonding network of water [17]. This effect is conceptually rooted in the Hofmeister series first reported in 1888 [18]. Since the observed effects exceed the ones known from classical chaotropic ions such as $I^{-}$ and ${\mathrm{SCN}}^{-}$ by far and set in at concentrations several orders of magnitude smaller, Naskar et al. [7] and Assaf et al. [19] coined the term “superchaotropes” for these large ions in 2015.

A question still under active debate remains: can nano‐ions be considered as classical electrolytes, despite their large size? Large water‐soluble POMs with sizes between 2 and 6 nm are known to self‐assemble into large hollow and spherical “blackberry” structures in dilute solutions [20], a behavior not expected for classical ions. Counterion‐mediated interactions and hydrogen bonds overcome the electrostatic repulsion in these systems. Similar self‐assembly into vesicles and small micelles has also been observed for smaller nano‐ions such as metallacarboranes, where aggregation arises from a delicate interplay of electrostatic, hydrophobic, and dispersion interactions [21]. For POMs with the Keggin structure [22] and sizes around 1 nm, molecular dynamics simulations revealed a possible aggregation into di‐, tri‐, and also higher oligomers consisting of a few ions each [23, 24]. Chaumont and Wipff predicted a transition from mainly hydrophobic forces at lower ion charge to interactions mediated by $H_{3}O^{+}$ counterions at higher charge [24]. Experimental evidence for these aggregation phenomena was found by Bera et al. using small angle x‐ray scattering in the bulk [25, 26] and at the air–water interface [27]. They described the interaction between the Keggin ions as long‐range repulsive due to electrostatic repulsion, but short‐range attractive due to hydrogen bonding, contradicting the expectations for classical ions [25].

Malinenko et al. addressed the question about the ionic or colloidal nature of Keggin ions from a different perspective and found a dual behavior [9]. On the one hand, Keggin ions adsorbed to the surface of non‐ionic surfactant micelles as (super)chaotropic anions. On the other hand, the diffusion coefficients and structure factors of dilute Keggin ion solutions with and without the addition of salt led to the contrary conclusion that their counterions can be neglected for the electrostatic repulsion between the Keggin ions themselves; a typical characteristic for colloidal systems. Drummond et al. demonstrated a strong adsorption of Keggin ions to surfaces of different wettability, both hydrophilic and hydrophobic interfaces [28]. In contrast, ${\mathrm{SCN}}^{-}$, a prototypical chaotropic anion, was excluded from hydrophilic surfaces. Therefore, the authors questioned the classification of Keggin ions as (super)chaotropic anions as a part of the extended Hofmeister series and suggested to treat them as small, soluble colloids.

Another promising approach to distinguish between ion‐like and colloid‐like behavior of charged particles is given by their influence on the electrostatic repulsion between charged interfaces, as captured by the inverse Debye screening length [29, 30, 31]:

(1) $$\kappa^{2}=\frac{2e^{2}I}{\varepsilon \varepsilon_{0}k_{B}T}.$$

For most classical electrolytes, both co‐ and counterions contribute fully to the electrostatic screening [32], with the exception of symmetric multivalent systems [33]. In contrast, for colloidal particles it has been shown that only the counterions govern the screening behavior, while the particles themselves form layers in parallel to the confining surfaces, leading to oscillatory structural forces [34, 35] and can be neglected in terms of electrostatic screening [35]. The same is valid for polyelectrolytes and their counterions [34, 36, 37, 38]. Braun et al. followed this idea and studied mixed foam films consisting of non‐ionic surfactants and Keggin ions with the thin film pressure balance (TFPB) [14]. They showed that the Keggin ions strongly adsorb at the interfaces of the foam films as chaotropic ions [39, 40], creating an electrostatic repulsion between both interfaces and an initial thickening of the films. At higher concentrations, the film thickness decreased again due to electrostatic screening by excess ions. A detailed analysis revealed that the Keggin ions showed a dual behavior with ion‐like properties (all ions contribute) at low concentrations and colloid‐like behavior (just the counterions contribute) at higher concentrations. However, the drawback of TFPB studies on these systems is that they do not provide access to pure Keggin ion solutions; surfactants are necessary to ensure the stability of the free‐standing foam films, which can alter the intrinsic behavior of the ions.

This limitation can be overcome by using colloidal probe atomic force microscopy (CP‐AFM), providing direct access to the electrostatic screening behavior without the necessity for further additives. CP‐AFM, as introduced independently by Butt [41] and Ducker et al. [42] in 1991, has proven to be a versatile tool to study the interactions between colloidal particles in the presence of various electrolytes. Since the beginning, interactions in monovalent electrolytes were investigated in detail [41, 42, 43, 44]. Further research was conducted on systems involving multivalent counter‐ [32, 45, 46, 47, 48, 49, 50] and coions [32, 45, 48, 50, 51], including symmetric multivalent electrolytes [33]. Many features of these interactions are still covered by the classical theory for the stability of colloidal dispersions as introduced by Derjaguin, Landau, Verwey, and Overbeek (DLVO theory) [52, 53, 54]. CP‐AFM has also been successfully applied to study other systems such as polyelectrolytes [55, 56, 57, 58], nanoparticles [59, 60, 61, 62, 63, 64] and micelles [60, 65, 66]. Importantly, by using negatively charged silica beads as probes, this technique minimizes the strong adsoption of Keggin ions observed in foam films, allowing us to focus our analysis on their screening effect.

With this work, we aim to shed light on the true nature of superchaotropic nano‐ions and their classification as ions, colloids or in between. Therefore, we chose the archetypical Keggin structure as a model system and provide the first systematic CP‐AFM study on aqueous solutions of phosphotungstate ${\left[{\mathrm{PW}}_{12}O_{40}\right]}^{3-}$ $\left({\mathrm{PW}}^{3-}\right)$ and silicotungstate ${\left[{\mathrm{SiW}}_{12}O_{40}\right]}^{4-}$ $\left({\mathrm{SiW}}^{4-}\right)$. Both possess an almost identical chemical structure and size, but different heteroatoms and charge densities, and therefore degrees of superchaotropicity. We cover a wide concentration range, with and without additional salt. From the measured interaction forces, numerical fits according to the well‐established DLVO theory enable the determination of the electrostatic screening behavior, which is more consistent with an ionic nature of the POMs, possibly involving aggregation, than with a colloidal character.

## 2 Results

We report colloidal probe AFM (CP‐AFM) measurements between silica microspheres in aqueous solutions of the two different polyoxometalates (POMs) with Keggin structure [22] phosphotungstate $\left({\mathrm{PW}}^{3-}\right)$ and silicotungstate $\left({\mathrm{SiW}}^{4-}\right)$. Both possess similar diameters of ≈ 1.1 to 1.2 nm [67, 68, 69], but a different charge and therefore charge density and degree of superchaotropicity. A visual representation of the two POMs is provided in Figure 1.

**FIGURE 1:**
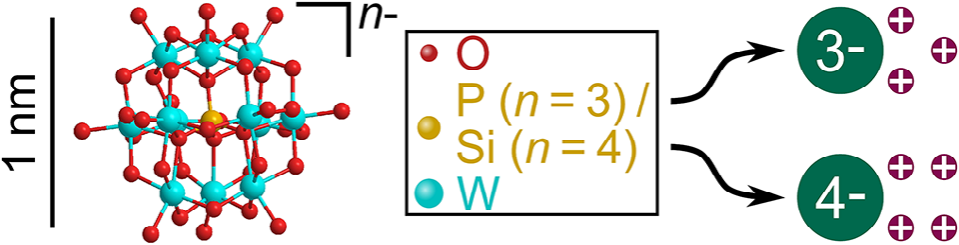
Chemical structure of the two Keggin‐type polyoxometalates (POMs) phosphotungstate ${\left[{\mathrm{PW}}_{12}O_{40}\right]}^{3-}$ $\left({\mathrm{PW}}^{3-}\right)$ and silicotungstate ${\left[{\mathrm{SiW}}_{12}O_{40}\right]}^{4-}$ $\left({\mathrm{SiW}}^{4-}\right)$. The type of heteroatom (phosphorus vs. silicon) modulates the charge density, number of counterions (three vs. four protons $H^{+}$ for ${\mathrm{PW}}^{3-}$ and ${\mathrm{SiW}}^{4-}$, respectively) and degree of superchaotropicity.

The first section 2.1 deals with the pure POM solutions. We compare the two ions at the same nominal ionic strength and model the data according to the DLVO theory to extract the properties of the charged silica surfaces and the screening behavior of the ions in solution. The second section 2.2 focuses on mixtures of ${\mathrm{SiW}}^{4-}$ with the monovalent salt NaCl. The additional salt alters the interactions between the POM ions and is expected to screen their electrostatic repulsion, consistent with previous bulk studies on POM‐POM structure factors [9]. Again, we apply the DLVO theory to extract the charging properties of the silica surfaces and the screening behavior of the ions in solution.

Across both cases, the employed model consists of the two contributions from the van der Waals (vdW) and the electric double layer (EDL) force

(2) $$F_{\mathrm{DLVO}}=F_{\mathrm{vdW}}+F_{\mathrm{EDL}},$$

c.f. Section S1.3. The Hamaker constant associated to the vdW force was determined for each pair of beads separately by using a 500 mM NaCl electrolyte to 0.6 × 10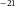 to 1.9 × 10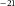 J (c.f. Table S3), in good agreement with the literature [33, 70]. It was then fixed to this value since the Hamaker constant is usually considered to be largely independent of the specific ion type [33]. This leaves the diffuse layer potential $\psi_{\mathrm{dl}}$, the ionic strength I of the solution and the regulation parameter p as free fit parameters for the numerical solution of the Poisson–Boltzmann (PB) Equation S4. The ionic strength was compared to its nominal value defined as

(3) $$I_{\mathrm{nom}}=\frac{1}{2}\sum_{i}c_{i,0}z_{i}^{2}=I_{\mathrm{nom}}^{\mathrm{POM}}+I_{\mathrm{nom}}^{\mathrm{NaCl}},$$

where the sum runs over all ionic species present, assuming that the contributions from the POMs and the added salt are additive. Under the investigated conditions, ${\mathrm{PW}}^{3-}$ and ${\mathrm{SiW}}^{4-}$ are fully dissociated (c.f. Section S1.1) and act as 1:z electrolytes with z=3 and z=4, respectively, accompanied by the corresponding number of counterions $H^{+}$. Both the POM anions at concentration $c_{0}$ and their counterions at concentration $c_{0}z$ are assumed to contribute to $I_{\mathrm{nom}}^{\mathrm{POM}}=1/2\left(c_{0}z+c_{0}z^{2}\right)$. All investigated solutions are summarized in Table S1.

### 2.1 Pure Polyoxometalate Solutions

First, the interaction forces across pure POM solutions were examined. The results for ${\mathrm{PW}}^{3-}$ and ${\mathrm{SiW}}^{4-}$ are presented in Figure 2a,b in a semi‐logarithmic representation to emphasize the electrostatic repulsion. The analysis is first restricted to nominal ionic strengths between 0.5 mM and 50 mM. These nominal ionic strengths were calculated from the known concentrations of the solutions according to Equation 3, assuming a complete dissociation of the POMs. The assumption of complete dissociation can be justified by the strong acidity of the Keggin structure and is confirmed by pH measurements (c.f. Sections S1.1 and S4.1) [5].

**FIGURE 2:**
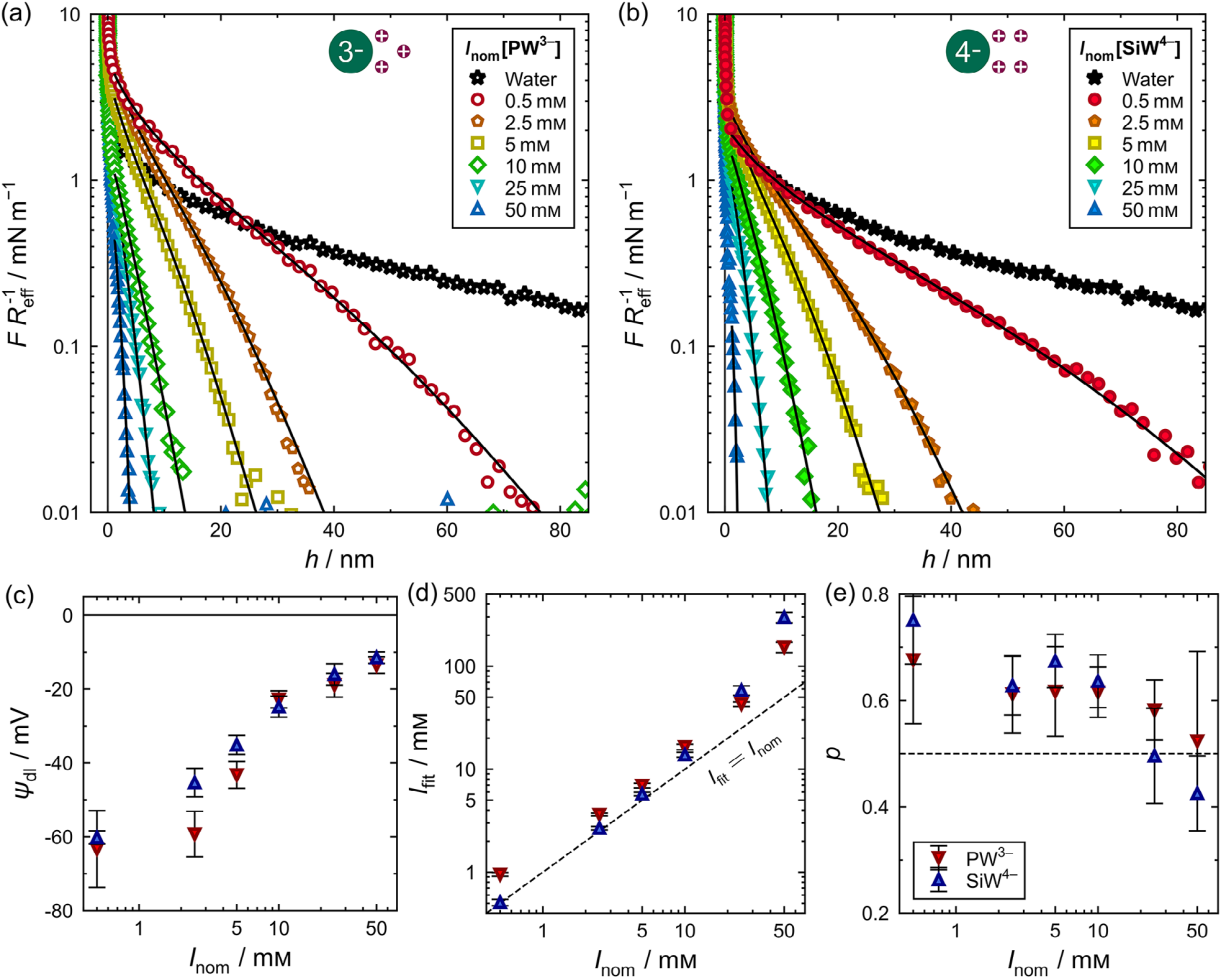
Interaction forces between silica beads in aqueous solutions of the two Keggin ions (a) ${\mathrm{PW}}^{3-}$ and (b) ${\mathrm{SiW}}^{4-}$. The legends refer to the nominal ionic strength of the solutions calculated according to Equation 3, the corresponding POM concentrations are given in Table S1. The colored symbols are data points, the black lines numerical fits according to the DLVO theory (Equation 2). A reference measurement in ultrapure water demonstrating long‐ranged repulsion is denoted by black stars. The corresponding fit parameters of the EDL force are displayed below: (c) diffuse layer potential $\psi_{\mathrm{dl}}$, (d) fitted ionic strength $I_{\mathrm{fit}}$ and (e) regulation parameter p in dependence on the nominal ionic strength $I_{\mathrm{nom}}$ of the two types of Keggin ions. The dashed line in (d) corresponds to the fitted ionic strength being equal to the nominal one, in (e) to p=0.5. An alternative representation of the fit parameters, based on the POM concentration instead of $I_{\mathrm{nom}}$, can be found in Figure S10.

Both systems exhibit an electrostatic repulsion, whose range decreases with increasing ionic strength of the solutions since the electrostatic screening becomes more effective. This behavior was already demonstrated for various different electrolytes [42, 43, 71, 72] and is not affected by the simultaneous change in pH [73]. The double layer repulsion, especially for the lower ionic strengths, exhibits a characteristic shape. In the semilogarithmic representation of Figure 2a,b, two almost linear regimes with different slopes are separated by a defined “kink” (e.g., at approximately 50–60 nm for 0.5 mM ${\mathrm{PW}}^{3-}$). This shape was found to be a common feature of multivalent coions and was traced back to the strong electrostatic repulsion between the negatively charged interfaces and the multivalent coions (i.e., Keggin ions) in the solution, leading to a depletion of the coions within the gap [50, 51]. Below a certain separation, where the “transition” occurs, the gap is completely depleted from the like‐charge coions, leaving only the counterions compensating for the surface charge. This reduces the effectiveness of the screening and leads to a shallower slope of the curve at small separations. At larger separations, both the counter‐ and coions are participating in the electrostatic screening and the decay can be described by the Debye‐Hückel approximation. The decay length therefore equals the Debye length $\kappa^{-1}$ from Equation 1 and is inversely proportional to $\sqrt{I}$.

The data were modeled according to the DLVO theory as denoted by the solid black lines in Figure 2a,b; the corresponding optimized model parameters are given in Figure 2c–e. Excellent agreement between data and fits is observed for both ions and all ionic strengths examined. Figure 2c shows the diffuse layer potential $\psi_{\mathrm{dl}}$. The symmetric setup provides only the magnitude of the potential ‐ not its sign. Since there are no indications of charge inversion, the sign is assumed to be negative throughout the entire concentration series, as is commonly observed for silica beads under alkaline and mildly acidic conditions [43, 74]. A detailed discussion of this assumption, including reference measurements with HCl to isolate the influence of pH, is provided in Section S4.2. The magnitude of $\psi_{\mathrm{dl}}$ decreases with increasing ionic strength, and this trend is nearly identical for both systems. Only at intermediate ionic strength does ${\mathrm{PW}}^{3-}$ yield a slightly more negative potential than ${\mathrm{SiW}}^{4-}$. This decrease in diffuse layer potential, despite the simultaneous increase in surface charge density (c.f. Section S4.3), is in line with previous observations for various electrolytes [32, 43, 44, 49, 73] and demonstrates that the behavior of $\psi_{\mathrm{dl}}$ is governed primarily by the increase in ionic strength rather than the increase in charge density.

Figure 2d displays the fitted ionic strength, $I_{\mathrm{fit}}$, for the ${\mathrm{PW}}^{3-}$ and ${\mathrm{SiW}}^{4-}$ solutions. For ${\mathrm{SiW}}^{4-}$, two distinct regimes are evident. At low ionic strengths $\left(I_{\mathrm{nom}}\leq 5\;\mathrm{mM}\right)$, $I_{\mathrm{fit}}$ is in excellent agreement with $I_{\mathrm{nom}}$. At higher ionic strengths $\left(I_{\mathrm{nom}}>5\;\mathrm{mM}\right)$, however, $I_{\mathrm{fit}}$ systematically exceeds $I_{\mathrm{nom}}$, indicating an electrostatic screening that is more effective than expected. The magnitude of this deviation increases with increasing ${\mathrm{SiW}}^{4-}$ concentration. ${\mathrm{PW}}^{3-}$ exhibits a comparable trend, although $I_{\mathrm{fit}}$ remains slightly higher than $I_{\mathrm{nom}}$ throughout the entire concentration range. The origin of the unexpectedly strong electrostatic screening observed in both systems is analyzed in detail in section 3.1.1.

Figure 2e depicts the regulation parameter p, locating the charge regulation properties of the silica beads between constant potential (CP, p=0) and constant charge (CC, p=1) conditions. For ${\mathrm{PW}}^{3-}$ it decreases from p≈0.68 to p≈0.52, for ${\mathrm{SiW}}^{4-}$ from p≈0.75 to p≈0.43 with increasing $I_{\mathrm{nom}}$. The observed decrease is slightly more pronounced for ${\mathrm{SiW}}^{4-}$, but in general both systems behave similarly. They evolve from a more CC‐like behavior (p>0.5) to a neither CC‐ nor CP‐like behavior (p≈0.5).

Up to this point, the ionic strength was constrained between 0.5 mM and 50 mM, as a distinct EDL repulsion is observed. However, at higher ionic strengths, this double layer repulsion vanishes. Figure 3 presents $I_{\mathrm{nom}}=100\;\mathrm{mM}$, which is a sufficiently high ionic strength for the interaction to become purely attractive for both systems. This attraction can be well‐described by the vdW force previously determined (details about the procedure are presented in Section S2). The black solid line follows Equation S3 with the Hamaker constant determined in 500 mM NaCl for the same combination of beads. Deviations from the vdW behavior are restricted to separations below 1nm, immediately before contact, and closely resemble those observed in 500 mM NaCl (c.f. Figure S5). These deviations are therefore attributed to surface roughness of the beads across the contact area rather than to any residual EDL contribution. Since simple electrolytes reproducibly exhibit EDL repulsion up to 100 mM ionic strength (c.f. Section S3), the absence of EDL repulsion for the POMs continues the aforementioned trend that the electrostatic screening is more effective than anticipated.

**FIGURE 3:**
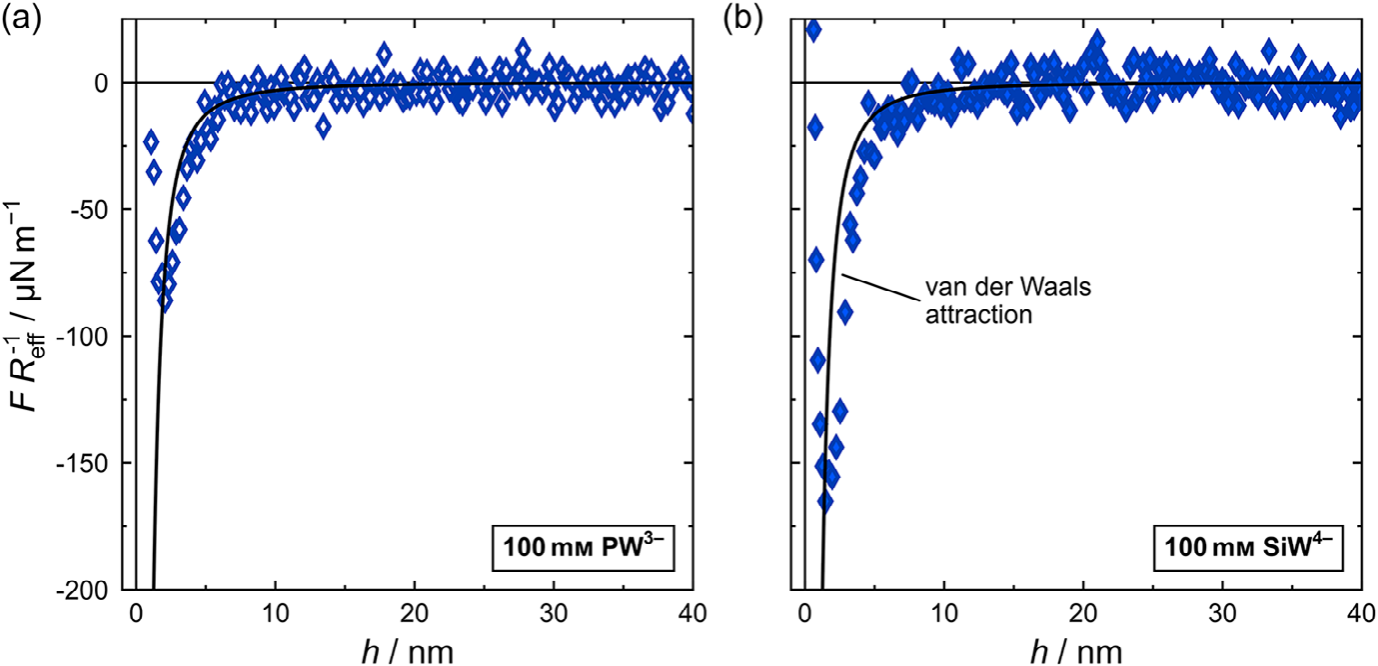
Interaction forces between silica beads in aqueous solutions of the two Keggin ions (a) ${\mathrm{PW}}^{3-}$ and (b) ${\mathrm{SiW}}^{4-}$ at a nominal ionic strength of 100 mM. The colored symbols are data points, the black lines denote the van der Waals force according to Equation S3 with the Hamaker constant from Table S3.

### 2.2 Solutions Containing Polyoxometalates and Salt

In this second section, we present the interaction forces across solutions containing ${\mathrm{SiW}}^{4-}$ and added NaCl. The results are shown in Figure 4a,b in semi‐logarithmic representation. We favored ${\mathrm{SiW}}^{4-}$ (instead of ${\mathrm{PW}}^{3-}$) for this analysis due to its superior hydrolytic stability [75]. Each solution contained either (a) $I_{\mathrm{nom}}^{{\mathrm{SiW}}^{4-}}=0.5\;\mathrm{mM}$ or (b) 5 mM of ${\mathrm{SiW}}^{4-}$ and a variable amount of NaCl. This corresponds to the two lowest ionic strengths studied in the previous section 2.1, where we demonstrated excellent agreement between $I_{\mathrm{nom}}$ and $I_{\mathrm{fit}}$ in Figure 2d. Both ionic strengths of ${\mathrm{SiW}}^{4-}$ show a similar behavior: with increasing NaCl concentration, the range of the repulsive EDL force decreases. Moreover, the overall range of the repulsion is significantly smaller for 5 mM than for 0.5 mM. This is in agreement with the expectations based on the Debye length (Equation 1), since the availability of more ions (i.e., salt or Keggin ions) should lead to a more effective screening within the double layer.

**FIGURE 4:**
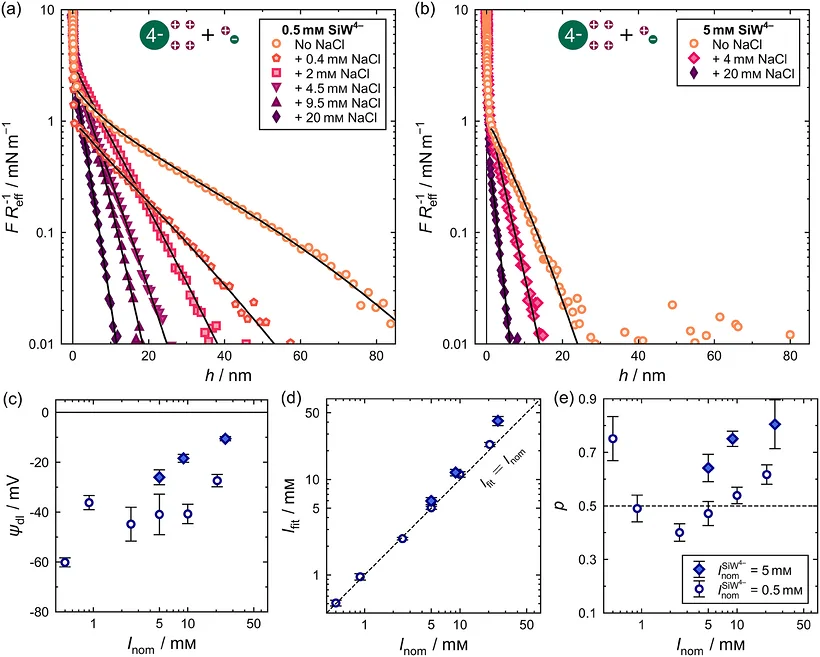
Interaction forces between silica beads in aqueous solutions containing (a) $I_{\mathrm{nom}}^{{\mathrm{SiW}}^{4-}}=0.5\;\mathrm{mM}$ or (b) 5 mM of ${\mathrm{SiW}}^{4-}$ and a variable amount of added salt NaCl. The colored symbols are data points, the black lines numerical fits according to the DLVO theory for fixed ionic strength of NaCl $\left(I_{\mathrm{fit}}=I_{\mathrm{nom}}^{\mathrm{NaCl}}+I_{\mathrm{fit}}^{{\mathrm{SiW}}^{4-}}\right)$. The corresponding fit parameters of the EDL force are displayed below: (c) the diffuse layer potential $\psi_{\mathrm{dl}}$, (d) the fitted ionic strength $I_{\mathrm{fit}}$ and (e) the regulation parameter p. For each $I_{\mathrm{nom}}^{{\mathrm{SiW}}^{4-}}$, $I_{\mathrm{nom}}$ was varied by the addition of salt, c.f. Equation 3 and Table S1. The dashed line in (d) corresponds to the fitted ionic strength being equal to the nominal one, in (e) to p=0.5. An alternative representation of the fit parameters, based on the NaCl concentration instead of $I_{\mathrm{nom}}$, can be found in Figure S11.

The data were again modeled according to the DLVO theory as denoted by the solid black lines in Figure 4a,b. The corresponding optimized model parameters are depicted in Figure 4c–e. The total nominal ionic strength $I_{\mathrm{nom}}$ is given by the sum of the contributions from ${\mathrm{SiW}}^{4-}$ and NaCl, $I_{\mathrm{nom}}=I_{\mathrm{nom}}^{{\mathrm{SiW}}^{4-}}+I_{\mathrm{nom}}^{\mathrm{NaCl}}$ (c.f. Equation 3). For given $I_{\mathrm{nom}}^{{\mathrm{SiW}}^{4-}}$, the variation in $I_{\mathrm{nom}}$ results from the addition of the respective amount of salt. A complete list of all investigated solutions is provided in Table S1.

Figure 4c presents the diffuse layer potential $\psi_{\mathrm{dl}}$ depending on $I_{\mathrm{nom}}$. $\psi_{\mathrm{dl}}$ is again assumed to be negative. Overall, it is more negative for 0.5 mM than for 5 mM of ${\mathrm{SiW}}^{4-}$. This aligns with $\psi_{\mathrm{dl}}$ in Figure 2c in the absence of salt; the addition of salt does not influence the order. However, for both $I_{\mathrm{nom}}^{{\mathrm{SiW}}^{4-}}$ (0.5 mM and 5 mM) the absolute value of $\psi_{\mathrm{dl}}$ decreases with increasing $I_{\mathrm{nom}}$. This again follows the common observation of a reduction in diffuse layer potential with increasing ionic strength [32, 43, 44, 49, 73].

Figure 4d displays the fitted ionic strength $I_{\mathrm{fit}}$. In our modeling, we assumed that the contributions from ${\mathrm{SiW}}^{4-}$ and NaCl to the ionic strength are additive, c.f. Equation 3. Furthermore, we fixed the contribution of NaCl to its nominal value, assuming that deviations between the observed screening behavior and the PB theory are caused by ${\mathrm{SiW}}^{4-}$ and not the salt. This is justified by multiple studies, demonstrating the near‐ideal screening behavior of NaCl over a wide concentration range [42, 43, 44]. Thus, the total fitted ionic strength is given by:

(4) $$I_{\mathrm{fit}}=I_{\mathrm{nom}}^{\mathrm{NaCl}}+I_{\mathrm{fit}}^{{\mathrm{SiW}}^{4-}}.$$

At $I_{\mathrm{nom}}^{{\mathrm{SiW}}^{4-}}=5\;\mathrm{mM}$ and in the absence of salt, $I_{\mathrm{fit}}$ agrees with the nominal ionic strength to within 20%. With increasing salt concentration, however, the fitted ionic strength exceeds the nominal value by up to 65%. A similar trend, but to a much smaller extent, is observed at $I_{\mathrm{nom}}^{{\mathrm{SiW}}^{4-}}=0.5\;\mathrm{mM}$. Thus, upon addition of salt we observe the same qualitative effect as in pure Keggin ion solutions at higher concentrations: the electrostatic screening is higher than expected. However, in the mixed system this enhanced screening is driven by the increased overall ionic strength introduced by the added salt rather than by an increased Keggin ion concentration.

Figure 4e depicts the regulation parameter p. At $I_{\mathrm{nom}}^{{\mathrm{SiW}}^{4-}}=0.5\;\mathrm{mM}$ the regulation parameter drops from p≈0.75 without salt (more CC‐like behavior) to a minimum of p≈0.40 at $I_{\mathrm{nom}}=2.5\;\mathrm{mM}$ (more CP‐like behavior). Upon further addition of salt, it increases to p≈0.62 again. At $I_{\mathrm{nom}}^{{\mathrm{SiW}}^{4-}}=5\;\mathrm{mM}$ there is no initial decrease. Instead, the regulation parameter increases monotonically with salt concentration from p≈0.64 (without salt) to p≈0.80 at $I_{\mathrm{nom}}=25\;\mathrm{mM}$, demonstrating a more CC‐like behavior over the examined concentration range.

## 3 Discussion

### 3.1 Pure Polyoxometalate Solutions

#### 3.1.1 Electrostatic Screening Behavior

Compared to classical ions, the two Keggin ions phosphotungstate $\left({\mathrm{PW}}^{3-}\right)$ and silicotungstate $\left({\mathrm{SiW}}^{4-}\right)$ are large in size whilst only carrying a few delocalized charges. Based on these two physical parameters, this places them between classical ions (typical diameter: 0.1nm−1nm, valence: 1to4) [76] and colloids (typical diameter: 1nm−1μm, high valence). Classical ions adhere to a Boltzmann distribution within the diffuse layer, meaning that they fully participate in the screening of surface charges [32]. In the case of an ion‐like behavior of the Keggin ions, the fitted ionic strength $I_{\mathrm{fit}}$ should therefore be equal to the nominal one $I_{\mathrm{nom}}$ according to Equation 3. This can be quantified via the ratio $I_{\mathrm{fit}}\;I_{\mathrm{nom}}^{-1}=1$. For common tri‐ and quadrivalent coions, a proper description of the interaction force by the DLVO theory down to distances of about 5nm and a good agreement between $I_{\mathrm{nom}}$ and $I_{\mathrm{fit}}$ were already demonstrated [32, 48, 50, 51]. This demonstrates that the populations of all co‐ and counterions still follow a Boltzmann distribution. In contrast, for larger colloidal particles with multiple charges, the particles are ordered layer‐wise parallel to the outer surfaces and can lead to oscillatory forces [34, 36, 37, 38]. Here, “colloidal” refers specifically to the electrostatic screening behavior: the particles themselves do not contribute directly to screening; rather, screening is provided only by their counterions and other small ions present in the system [35], resulting in an ionic strength of

(5) $$\begin{array}{cc} I_{\mathrm{colloid}} & =1.5\cdot c_{{\mathrm{PW}}^{3-}}\;\;\text{for}\;{\mathrm{PW}}^{3-} \end{array}$$

(6) $$\begin{array}{cc} I_{\mathrm{colloid}} & =2\cdot c_{{\mathrm{SiW}}^{4-}}\;\;\text{for}\;{\mathrm{SiW}}^{4-}. \end{array}$$

Therefore, in the case of a colloid‐like behavior of the Keggin ions, the electrostatic screening should become significantly less effective than for an ion‐like behavior. An earlier study by Braun et al. on mixtures between a non‐ionic surfactant and ${\mathrm{SiW}}^{4-}$ locates ${\mathrm{SiW}}^{4-}$ between these two limiting cases of ions and colloids, while the exact position depends on the ionic strength of the solution [14]. At $I_{\mathrm{nom}}=0.5\;\mathrm{mM}$, the behavior of ${\mathrm{SiW}}^{4-}$ is reported to resemble the one of a classical ion, to be intermediate at $I_{\mathrm{nom}}=5\;\mathrm{mM}$ and to become colloid‐like at $I_{\mathrm{nom}}=25\;\mathrm{mM}$.

The results presented here suggest a different behavior for both polyoxometalates (POMs), ${\mathrm{PW}}^{3-}$ and ${\mathrm{SiW}}^{4-}$. Figure 5a highlights ion‐like behavior by showing the ratio $I_{\mathrm{fit}}\;I_{\mathrm{nom}}^{-1}$ (with data taken from Figure 2d). In contrast, Figure 5b emphasizes colloid‐like behavior by displaying the ratio $I_{\mathrm{fit}}\;I_{\mathrm{colloid}}^{-1}$ according to Equations (5), (6). Accordingly, $I_{\mathrm{fit}}\;I_{\mathrm{nom}}^{-1}=1$ in Figure 5a would indicate ideal ion‐like behavior, whereas $I_{\mathrm{fit}}\;I_{\mathrm{colloid}}^{-1}=1$ in Figure 5b would correspond to colloid‐like behavior. In both representations, two clearly separated regimes are observed.

**FIGURE 5:**
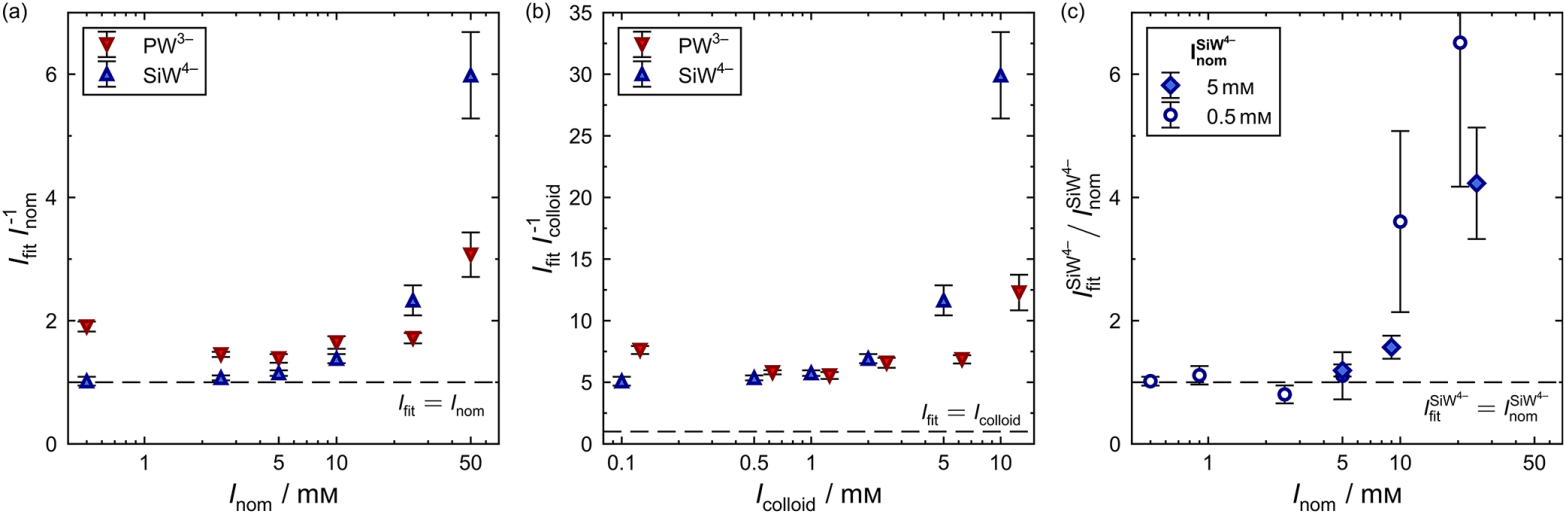
(a) & (b) Normalized ionic strength for the pure ${\mathrm{PW}}^{3-}$ and ${\mathrm{SiW}}^{4-}$ solutions. In (a) ion‐like behavior was assumed and $I_{\mathrm{fit}}\;I_{\mathrm{nom}}^{-1}$ was calculated from the fitted ionic strength from Figure 2d and the nominal value according to Equation 3. In (b), the assumption of colloid‐like behavior of the POMs allowed to calculate $I_{\mathrm{fit}}\;I_{\mathrm{colloid}}^{-1}$ according to Equations (5), (6). The dashed lines correspond to ideal ion‐like and colloid‐like behavior, respectively. (c) Normalized ionic strength of ${\mathrm{SiW}}^{4-}$ for the mixed ${\mathrm{SiW}}^{4-}$ and NaCl solutions. $I_{\text{fit}}^{{\mathrm{SiW}}^{4-}}$ was extracted from the data presented in Figure 4d via Equation 4, $I_{\text{nom}}^{{\mathrm{SiW}}^{4-}}$ calculated according to Equation 3. The dashed line corresponds to the fitted ionic strength being equal to the nominal one.

For $I_{\mathrm{nom}}$ up to 5 mM, the behavior of ${\mathrm{PW}}^{3-}$ and ${\mathrm{SiW}}^{4-}$ in Figure 5a diverges. For ${\mathrm{PW}}^{3-}$, the electrostatic screening is more effective than predicted for simple ions $\left(I_{\mathrm{fit}}\;I_{\mathrm{nom}}^{-1}>1\right)$. We attribute this deviation to the formation of more highly charged fragments (c.f. Section S1.1). ${\mathrm{PW}}^{3-}$ is only stable under highly acidic conditions and is partially hydrolyzed into the monolacunary species ${\mathrm{PW}}_{11}O_{39}^{7-}$ at low concentrations (corresponding to *pH ≳ 2)* [75, 77]. With increasing $I_{\mathrm{nom}}$, the pH of the solutions decreases (c.f. Section S4.1), which enhances the hydrolytic stability and shifts ${\mathrm{PW}}^{3-}$ toward ion‐like behavior. Based on hydrolytic stability alone, we would therefore expect a monotonic decrease of $I_{\mathrm{fit}}\;I_{\mathrm{nom}}^{-1}$, approaching unity at high $I_{\mathrm{nom}}$. In contrast, ${\mathrm{SiW}}^{4-}$ is substantially more stable within the investigated pH range, rendering decomposition effects unlikely [75]. Consistent with this stability, ${\mathrm{SiW}}^{4-}$ exhibits ion‐like behavior $\left(I_{\mathrm{fit}}\;I_{\mathrm{nom}}^{-1}\approx 1\right)$ in this concentration regime. Despite differences in absolute values, the same qualitative trends are reflected in Figure 5b. However, for both systems the ratio $I_{\mathrm{fit}}\;I_{\mathrm{colloid}}^{-1}$ exceeds unity by several times, demonstrating that a colloid‐like description does not hold for either Keggin ion.

Above $I_{\mathrm{nom}}=5\;\mathrm{mM}$, the simultaneous increase of $I_{\mathrm{fit}}\;I_{\mathrm{nom}}^{-1}$ in Figure 5a for both POMs indicates the onset of a different mechanism. In this concentration regime, the experimental observations for ${\mathrm{SiW}}^{4-}$ can no longer be adequately described by classical ion‐like screening models, which predict weaker electrostatic screening than experimentally observed (c.f. Figure 5a). At the same time, a colloid‐like description (c.f. Figure 5b) does not provide an improved representation of the data for either POM. Instead, an unexpectedly strong electrostatic screening is observed, which is more pronounced for ${\mathrm{SiW}}^{4-}$ than for ${\mathrm{PW}}^{3-}$ and cannot be explained solely by the nominal solution concentrations and compositions. We propose that this enhanced electrostatic screening originates from ion self‐aggregation, leading to charged species with effective valences greater than 3 and 4, respectively. In the following, we therefore restrict our analysis to a modified ion‐like description that incorporates ion aggregation, demonstrating that the data remain consistent with an ionic nature of the POMs.

#### 3.1.2 Aggregation

The proposed interpretation of the unexpectedly strong electrostatic screening as a manifestation of ion aggregation is supported by the unique properties of superchaotropic Keggin ions. Their small charge density reduces the electrostatic barrier arising from Coulomb repulsion between like‐charged ions, while their large size and high polarizability [17] promote attractive dispersion interactions [31, 78]. This makes it plausible that the dispersion interactions are able to overcome the electrostatic repulsion, thereby facilitating aggregation.

The average aggregate size can be estimated from $I_{\mathrm{fit}}$ using a simplified model. We denote the initial concentration of Keggin ions as $c_{0}$. Since ${\mathrm{PW}}^{3-}$ and ${\mathrm{SiW}}^{4-}$ are 1:z electrolytes with z=3 and z=4, respectively, this corresponds to a counterion $\left(H^{+}\right)$ concentration of $c_{H^{+}}=c_{0}z$. Upon dissolution in water, the non‐aggregated ions are replaced by a distribution of Keggin ion aggregates characterized by aggregation numbers ${\left(n_{i}\right)}_{i\geq 1}$ and corresponding concentrations ${\left(c_{i}\right)}_{i\geq 1}$. Particle number conservation requires $c_{0}=\sum_{i\geq 1}c_{i}n_{i}$ to hold. The distribution of aggregation numbers and the corresponding concentrations are unknown and not easily examinable, so some simplifying assumptions are made in the analysis. Herein, we assume that all solutions are binary, i.e., contain only aggregates of one type and their counterions. This implies that the distribution of different aggregation numbers is replaced by a single apparent aggregation number $n_{\mathrm{agg}}\geq 1$, creating the same ionic strength of the solution. Compared to the isolated ions at concentration $c_{0}$, the concentration of aggregates is reduced to $c_{\mathrm{agg}}=c_{0}/n_{\mathrm{agg}}$, while the concentration of monovalent counterions $c_{H^{+}}$ is not affected by the aggregation. Each aggregate carries a charge of $z_{\mathrm{agg}}=-n_{\mathrm{agg}}z$. According to Equation 3, the higher charge per charge carrier overcompensates their reduced concentration, leading to an increased ionic strength of

(7) $$\begin{array}{cc} I_{\mathrm{fit}} & =\frac{1}{2}\left[c_{H^{+}}+c_{\mathrm{agg}}\cdot z_{\mathrm{agg}}^{2}\right]=\frac{1}{2}\left[c_{0}z+\frac{c_{0}}{n_{\mathrm{agg}}}{\left(n_{\mathrm{agg}}z\right)}^{2}\right]\\ & =\frac{c_{0}z}{2}\left(1+n_{\mathrm{agg}}z\right). \end{array}$$

We retrieve $I_{\mathrm{nom}}$, the case without an aggregation, by setting $n_{\mathrm{agg}}=1$. Like this, the apparent aggregation number can be determined via

(8) $$n_{\mathrm{agg}}=\frac{\left(1+z\right)I_{\mathrm{fit}}\;I_{\mathrm{nom}}^{-1}-1}{z}.$$

Table 1 shows the apparent aggregation numbers as calculated from the data in Figure 5a, which are considered as mean aggregation numbers.

**TABLE 1:** Apparent aggregation numbers $n_{\mathrm{agg}}$ for the two types of Keggin ions. Calculated from $I_{\mathrm{fit}}\;I_{\mathrm{nom}}^{-1}$ as given in Figure 5a using Equation 8.

$I_{\mathrm{nom}}$	$n_{\mathrm{agg}}^{{\mathrm{PW}}^{3-}}$	$n_{\mathrm{agg}}^{{\mathrm{SiW}}^{4-}}$
0.5mM	2.2	1.0
2.5mM	1.6	1.1
5.0mM	1.5	1.2
10.0mM	1.9	1.5
25.0mM	2.0	2.7
50.0mM	3.8	7.2

For ${\mathrm{PW}}^{3-}$, $n_{\mathrm{agg}}$ ranges between 1.5 and 3.8, indicating that aggregates of up to four ions are necessary to account for the unexpectedly strong electrostatic screening that is observed. This is consistent with MD simulations by Chaumont and Wipff, which predict a significant formation of di‐, tri‐, and larger oligomers for ${\mathrm{PW}}^{3-}$, mainly at $c^{{\mathrm{PW}}^{3-}}\geq 100\;\mathrm{mM}$ [23, 24]. The fraction of aggregated ions in their MD simulations depends on the counterion with 32.6% and 46.5% for $H_{3}O^{+}$ and $H_{5}O_{2}^{+}$, respectively, at $c^{{\mathrm{PW}}^{3-}}=150\;\mathrm{mM}$ [23]. For $H_{3}O^{+}$, the fraction of aggregated ions increases from just 15.7% at $c^{{\mathrm{PW}}^{3-}}=79\;\mathrm{mM}$ to 87.3% at 428 mM [24]. MD simulations by Bera et al., modeling ${\mathrm{PW}}^{3-}$ in the presence of 100 mM HCl, confirm this trend. They predict 30.2% of aggregated ions at $c^{{\mathrm{PW}}^{3-}}=60\;\mathrm{mM}$ and 74.9% at 200 mM [25]. All studies have in common that the tendency to aggregate increases with the concentration, but the probability to find an aggregate with a certain number of ions decreases monotonically with its size. This excludes monodisperse aggregates and shows that under most conditions the fraction of aggregates consisting of more than 4 ions is negligible, which is in agreement with our estimations. However, experimental data point toward even larger aggregates. DLS data by Hohenschutz et al. show hydrodynamic radii of ${\mathrm{PW}}^{3-}$ aggregates of around 35nm at $c^{{\mathrm{PW}}^{3-}}=100\;\mathrm{mM}$ [4]. Considering ion diameters of ≈ 1.1 to 1.2 nm [67, 68, 69], these aggregates have to consist of tens of ions each.

Notably, our data reveal that the unexpectedly strong electrostatic screening is significantly more pronounced for the more highly charged ${\mathrm{SiW}}^{4-}$ than for the less charged ${\mathrm{PW}}^{3-}$ under confinement, despite the stronger electrostatic repulsion. For ${\mathrm{SiW}}^{4-}$, $n_{\mathrm{agg}}$ ranges from 1.0 to 7.2, indicating larger aggregate sizes than those calculated for ${\mathrm{PW}}^{3-}$. This finding contrasts with previous literature reports, which investigated bulk systems. MD simulations by Chaumont and Wipff, for example, show a stronger aggregation tendency for ${\mathrm{PW}}^{3-}$ than for ${\mathrm{SiW}}^{4-}$ [24]. At the highest investigated concentration of 388 mM for instance, they find 45.4% of aggregated ions for ${\mathrm{SiW}}^{4-}$, but 87.3% for ${\mathrm{PW}}^{3-}$. Moreover, the average aggregate size is significantly larger for ${\mathrm{PW}}^{3-}$ than for ${\mathrm{SiW}}^{4-}$. Experimentally, no substantial aggregation of ${\mathrm{SiW}}^{4-}$ has been reported previously [4, 25]. Therefore, to our knowledge, the present work provides the first experimental evidence of significant aggregation in ${\mathrm{SiW}}^{4-}$ solutions. This suggests that Keggin ion aggregation is sensitive to environmental conditions, and that confinement might be able to fundamentally alter their aggregation behavior compared to bulk systems.

Our preceding analysis demonstrates that self‐aggregation of Keggin ions is described both theoretically and experimentally in literature [4, 23, 24, 25]. In agreement with our observations, the aggregation becomes more pronounced with increasing ionic strength [23, 24, 25]. An elevated ionic strength effectively screens the charge of the aggregates, thereby reducing the electrostatic barrier to their formation. Despite this well‐established trend, a clear paucity exists in the understanding of Keggin ion aggregation in the mM range. A substantial aggregation has primarily been found at high Keggin ion concentrations (≥100mM). In contrast, our experiments were conducted below 20 mM (c.f. Table S1), limited by the strong screening effects of multivalent ions. Beyond this threshold, the double layer repulsion is fully screened and thus no longer measurable. We hypothesize that the unexpectedly strong electrostatic screening observed in our system arises from the strong confinement imposed by the silica beads. MD simulations have emphasized the importance of protons (e.g., as $H_{3}O^{+}$ and $H_{5}O_{2}^{+}$) in mediating the Keggin ion aggregation [23, 24, 25]. Protons are attracted to the negatively charged silica surfaces, accumulating within the gap following a Boltzmann distribution. This elevated proton concentration compared to the bulk might facilitate the formation of proton‐bridged hydrogen bonds between Keggin ions, thereby promoting an aggregation at lower bulk concentrations.

### 3.2 Role of Salt

#### 3.2.1 Electrostatic Screening Behavior

Electrostatic repulsion between like‐charged ions plays a major role in preventing their aggregation by keeping the ions apart. To enable self‐aggregation, this long‐range repulsion between the Keggin ions must be overcome by short‐range attractive forces, such as dispersion forces, hydrophobic interactions, and water‐ or proton‐bridged hydrogen bonds [24, 25]. Consequently, the ionic strength of the solution is expected to strongly influence the aggregation by screening the electrostatic repulsion between the negatively charged Keggin ions, thereby facilitating close ion–ion contacts. A straightforward way to vary the ionic strength, and thus the interionic interactions, without altering the concentration of Keggin ions is the addition of a background electrolyte. Herein, NaCl served this purpose.

Recall that within our simplified model the fitted ionic strength of solutions containing ${\mathrm{SiW}}^{4-}$ and NaCl is given by Equation 4. We focus our following analysis on the Keggin ion contribution $I_{\mathrm{fit}}^{{\mathrm{SiW}}^{4-}}$. As shown in Figure 5c, the addition of NaCl induces a comparable enhancement of electrostatic screening in ${\mathrm{SiW}}^{4-}$ solutions to that observed in the absence of salt. This observation suggests that the dependence on ionic strength, already observed in Figure 5a, is to some extent universal, governed primarily by the screening of electrostatic repulsion. This finding is in line with MD simulations conducted by Chaumont and Wipff, predicting only minor changes in the extent of aggregation when $H_{3}O^{+}$ is replaced by ${\mathrm{Na}}^{+}$ in the case of ${\mathrm{SiW}}^{4-}$ [24], and supports our hypothesis of a self‐aggregation driven by screened electrostatic repulsion. The unexpectedly strong electrostatic screening seems to be more pronounced for the lower ${\mathrm{SiW}}^{4-}$ content than for the higher one, possibly due to the larger number of salt ions available per Keggin ion.

#### 3.2.2 Aggregation

We followed the approach described in section 3.1.2 for pure polyoxometalate solutions to calculate the apparent aggregation numbers $n_{\mathrm{agg}}^{{\mathrm{SiW}}^{4-}}$ for the mixed system consisting of ${\mathrm{SiW}}^{4-}$ and NaCl. Therefore, Equation 8 was adapted to

(9) $$n_{\mathrm{agg}}=\frac{\left(1+z\right)I_{\mathrm{fit}}^{{\mathrm{SiW}}^{4-}}/\;I_{\mathrm{nom}}^{{\mathrm{SiW}}^{4-}}-1}{z}.$$

The resulting aggregate sizes calculated from the data displayed in Figure 5c are presented in Table 2. For both ionic strengths of ${\mathrm{SiW}}^{4-}$, two distinct regimes can be identified. Up to $I_{\mathrm{nom}}=5\;\mathrm{mM}$, $n_{\mathrm{agg}}^{{\mathrm{SiW}}^{4-}}$ remains close to 1, indicating that the ${\mathrm{SiW}}^{4-}$ ions exist predominantly as isolated species at low salt concentrations. At higher ionic strength $\left(I_{\mathrm{nom}}>5\;\mathrm{mM}\right)$, corresponding to larger amounts of added salt, $n_{\mathrm{agg}}^{{\mathrm{SiW}}^{4-}}$ increases significantly. In line with our observation in the previous section, this increase is more pronounced for the lower ${\mathrm{SiW}}^{4-}$ content. Here, we hypothesize that the relative number densities of POM to NaCl ions play a dominant role, and suggest that a higher concentration of NaCl is required to achieve an analogous impact on $n_{\mathrm{agg}}^{{\mathrm{SiW}}^{4-}}$ for the higher ${\mathrm{SiW}}^{4-}$ content.

**TABLE 2:** Apparent aggregation numbers $n_{\mathrm{agg}}^{{\mathrm{SiW}}^{4-}}$ for the two lowest nominal ionic strengths of ${\mathrm{SiW}}^{4-}$ $\left(I_{\mathrm{nom}}^{{\mathrm{SiW}}^{4-}}=0.5\;\mathrm{mM}\;\mathrm{and}\;5\;\mathrm{mM}\right)$ with added salt. Calculated from $I_{\text{fit}}^{{\mathrm{SiW}}^{4-}}/\;I_{\text{nom}}^{{\mathrm{SiW}}^{4-}}$ as depicted in Figure 5c using Equation 9.

$I_{\mathrm{nom}}$	$n_{\mathrm{agg}}^{{\mathrm{SiW}}^{4-}}$	
	0.5 mM	5 mM
0.5 mM	1.0 ^a^	/
0.9 mM	1.1	/
2.5mM	0.8	/
5.0 mM	1.1	1.2 ^a^
9.0 mM	/	1.7
10.0 mM	4.3	/
20.5 mM	7.9	/
25.0 mM	/	5.4

^a^ Pure Keggin ion solutions (c.f. Table S1).

Although the absolute values differ, the qualitative trend of $n_{\mathrm{agg}}^{{\mathrm{SiW}}^{4-}}$ in the mixed systems closely mirrors the behavior observed for pure POMs as shown in Table 1. These findings suggest a transition near $I_{\mathrm{nom}}=5\;\mathrm{mM}$ from isolated ions, dominated by repulsive interactions, to ionic aggregates stabilized by attractive forces. This transition seems to occur regardless of whether the ionic strength arises solely from POMs or is created by a background electrolyte.

## 4 Conclusion

This study emphasizes the distinctive properties of nanometric, multivalent ions in aqueous solutions. Colloidal probe atomic force microscopy (CP‐AFM) was employed to investigate the interaction forces between charged silica microspheres across aqueous solutions of two polyoxometalates (POMs). The two POMs with the Keggin structure, phosphotungstate ${\left[{\mathrm{PW}}_{12}O_{40}\right]}^{3-}$ $\left({\mathrm{PW}}^{3-}\right)$ and silicotungstate ${\left[{\mathrm{SiW}}_{12}O_{40}\right]}^{4-}$ $\left({\mathrm{SiW}}^{4-}\right)$, were studied with and without added NaCl as a background electrolyte. The CP‐AFM data were modeled according to the DLVO theory, taking into account the electric double layer and van der Waals contributions in order to extract the electrostatic screening behavior of the POMs. Specifically, we asked whether all ions contribute to electrostatic screening, as expected for an *ion‐like behavior* of the POMs, or whether screening is governed solely by the small counterions, excluding the POM ions themselves, which would indicate a *colloid‐like behavior*.

Above a certain concentration, electrostatic screening becomes significantly stronger than expected for both types of POMs, which we ascribe to ion aggregation. These results are more consistent with an ionic nature of the POMs than with a colloidal character. The enhanced screening, and thus the aggregation tendency, appears to be more pronounced for the higher charged ${\mathrm{SiW}}^{4-}$ than for ${\mathrm{PW}}^{3-}$. This observation conflicts with earlier findings from literature for bulk solutions, where the stronger aggregation was found for ${\mathrm{PW}}^{3-}$ [24, 25]. We hypothesize that this difference may originate from the strong confinement imposed by the silica beads, which could alter the aggregation behavior of the POMs compared to the bulk. Additional experiments on mixed systems containing ${\mathrm{SiW}}^{4-}$ and NaCl show that the addition of NaCl to low concentrated solutions of ${\mathrm{SiW}}^{4-}$ (up to an ionic strength of 5 mM) gives rise to similarly enhanced electrostatic screening as for the pure POM solutions at higher concentrations. The salt promotes the POM aggregation by screening the electrostatic repulsion between like‐charged ions. This further substantiates the hypothesis that the enhanced screening arises from ionic aggregates.

The consistent observation of unexpectedly strong electrostatic screening across both pure and mixed systems strongly suggests the formation of POM aggregates, particularly for ${\mathrm{SiW}}^{4-}$. In contrast to earlier investigations conducted exclusively in bulk solutions, our confined‐geometry experiments provide, to the best of our knowledge, the first experimental evidence for ion aggregation in ${\mathrm{SiW}}^{4-}$ systems. Our research paves the way for similar investigations on other nanometric ions that were classified as superchaotropes in literature before, such as dodecaborate clusters.

## Conflicts of Interest

The authors declare no conflicts of interest.

## Supporting information


**Supporting File**: anie73627‐sup‐0001‐SuppMat.pdf.

## Data Availability

The data that support the findings of this study are openly available in TUdatalib at https://doi.org/10.48328/tudatalib‐1925.
