# Supplementary material for: Unexpectedly Strong Electrostatic Screening in Nano‐Ion Solutions
Source: Angew Chem Int Ed Engl. 2026 Aug 20;65(42):e1870244. doi: 10.1002/anie.1870244 (PMC13653632; doi:10.1002/anie.1870244)
Supplement: Supplementary file 1 [file ANIE-65-e1870244-s001.pdf]

Supporting Information: Unexpectedly Strong Electrostatic Screening in Nano-Ion Solutions

Thomas Tilger, Esther Ohnesorge, Hayden Robertson, Michael Ludwig, Michalis Tsintsaris, and Regine von Klitzing*

Soft Matter at Interfaces, Department of Physics, Technical University of Darmstadt, D-64289 Darmstadt, Germany

E-mail: klitzing@smi.tu-darmstadt.de

Contents

S1 Experimental Section **S-3**

S1.1 Materials S-3

S1.2 Methods S-5

S1.2.1 Preparation of the Substrate and Colloidal Probe S-5

S1.2.2 Characterization of the Silica Beads S-5

S1.2.3 Colloidal Probe Atomic Force Microscopy (CP-AFM) S-8

S1.3 Electrostatic Modeling S-9

S1.3.1 Van der Waals (vdW) Force S-9

S1.3.2 Electric Double Layer (EDL) Force S-10

S2 Determination of the Hamaker Constant **S-13**

S3 Polyoxometalates and NaCl at 100 mM **S-15**

S4 Surface Charge Density **S-16**

S4.1 pH Measurements S-16

S4.2 Sign of the Surface Charge S-17

S4.3 Pure Polyoxometalates S-20

S4.4 Polyoxometalates with Salt S-21

S5 Fit Parameters: Alternative Representations **S-23**

S5.1 Pure Polyoxometalates S-23

S5.2 Polyoxometalates with Salt S-23

S6 Electrical Conductivity Measurements **S-25**

References **S-28**

S1 Experimental Section

S1.1 Materials

The two heteropolyacids (HPAs) with the Keggin structure [1] phosphotungstic acid hydrate ($H_3PW_{12}O_{40} \cdot nH_2O$, for analysis EMSURE®) and silicotungstic acid hydrate ($H_4SiW_{12}O_{40} \cdot mH_2O$, for analysis EMSURE®), along with sodium chloride (NaCl, Supelco® 99.99 % Suprapur®), were purchased from Merck KGaA (Darmstadt, Germany). The phosphotungstic acid hydrate was placed in a container evacuated to ≈ 4 mbar at 25 °C for 3 h to obtain the hexahydrate [2, 3] and the silicotungstic acid hydrate heated to 350 °C at ambient pressure for 2 h to obtain the anhydrous form [4]. A longer duration did not lead to any additional decrease in weight. The initial water content of the two HPAs was then derived from the mass loss during drying to $n = 16.5$ and $m = 24.2$. The weight loss of NaCl during heating was negligible, therefore it was assumed to be anhydrous.

All chemicals were used as received, without further purification. The solutions were prepared from the hydrated HPAs using ultrapure water (Milli-Q® IQ 7000, 18.2 MΩ cm resistivity at 25 °C, Merck KGaA, Darmstadt, Germany), with the previously determined water content taken into account. Prior to use, the glassware was cleaned by soaking for at least 1 h in 1 % Hellmanex® III (Hellma GmbH & Co. KG, Müllheim, Germany), followed by thorough rinsing with copious amounts of ultrapure water. Table S1 summarizes all investigated solutions; a detailed characterization, including electrical conductivity and pH measurements, is provided in Sections S4.1 and S6. Both HPAs are strongly acidic. $H_3PW_{12}O_{40}$ is classified as a *superacid* [5]. The acidity of $H_4SiW_{12}O_{40}$ is weaker [6–8], yet it still behaves as a tetrabasic acid with all $pK_a < 2$ [9]. This pronounced acidity, stronger than that of normal inorganic acids, arises from a low charge density and significant charge delocalization [10–13]. Under the investigated conditions, the HPAs fully dissociate, yielding the polyoxometalates (POMs) phosphotungstate $\left([PW_{12}O_{40}]^{3-}\right.$, abbreviation: $\left.PW^{3-}\right)$ and silicotungstate $\left([SiW_{12}O_{40}]^{4-}\right.$, abbreviation: $\left.SiW^{4-}\right)$, respectively, together with the corresponding

Table S1: List of the investigated solutions. The nominal ionic strength I_{nom} was calculated according to Equation (S6) as the sum of the contributions from the POMs and NaCl.

Type of Solution	c^{POM} mM	I_{nom}^{POM} mM	c^{NaCl} mM	I_{nom}^{NaCl} mM	I_{nom} mM
PW^{3-}	0.08	0.5	/	/	0.5
	0.42	2.5	/	/	2.5
	0.83	5	/	/	5
	1.67	10	/	/	10
	4.17	25	/	/	25
	8.33	50	/	/	50
	16.67	100	/	/	100
SiW^{4-}	0.05	0.5	/	/	0.5
	0.25	2.5	/	/	2.5
	0.50	5	/	/	5
	1.00	10	/	/	10
	2.50	25	/	/	25
	5.00	50	/	/	50
	10.00	100	/	/	100
SiW^{4-} + NaCl	0.05	0.5	0.4	0.4	0.9
			2.0	2.0	2.5
			4.5	4.5	5
			9.5	9.5	10
			20.0	20.0	20.5
SiW^{4-} + NaCl	0.50	5	4.0	4.0	9
			20.0	20.0	25

number of protons. Accordingly, the pH of the POM solutions decreased monotonically with increasing POM concentration, from 3.3 to 1.0 ($c_{PW^{3-}}$ = 0.08 mM to 16.67 mM) for PW^{3-} and from 3.0 to 1.2 ($c_{SiW^{4-}}$ = 0.05 mM to 10 mM) for SiW^{4-}. Closely correlated with the pH is the hydrolytic stability. PW^{3-} is only stable under acidic conditions and decomposes into several fragments with increasing pH [14–17]. As main species below pH = 3.5 the ions $P_2W_{21}O_{71}^{6-}$, $PW_{11}O_{39}^{7-}$, $P_2W_{18}O_{62}^{6-}$, $P_2W_{19}O_{67}^{10-}$ and $P_2W_{20}O_{70}^{10-}$ are expected [15–17]. The implications of this partial decomposition for our results are discussed in section . By contrast, SiW^{4-} is significantly more stable than PW^{3-} within the investigated pH range [14, 18–21], and decomposition is not expected to affect the present results.

S1.2 Methods

S1.2.1 Preparation of the Substrate and Colloidal Probe

Silica microspheres with a nominal mean diameter of $(5.0 \pm 0.5)\,\mu m$ were purchased from Bangs Laboratories, Inc. (Fishers, USA), a tipless cantilever HQ:CSC37/tipless/No Al from Innovative Solutions Bulgaria Ltd. (Sofia, Bulgaria) and a polished quartz glass disk from Plano GmbH (Wetzlar, Germany). The quartz disk was cleaned in a 3 : 1 (vol:vol) piranha solution with 3 parts of sulfuric acid (ROTIPURAN® 98 %) and 1 part of hydrogen peroxide (30 %, ROTIPURAN® p.a., both from Carl Roth GmbH + Co. KG, Karlsruhe, Germany) for 30 min to remove any organic contaminations. Then the silica beads were sprinkled on the clean disk and sintered at 1150 °C for 3 h to firmly attach them. The tipless cantilever (HQ:CSC37/tipless/No Al, $f_{res} \approx 20\,kHz$, $k \approx 0.3\,N\,m^{-1}$) was cleaned in an oxygen plasma (pressure: 0.4 mbar, power: 50 W, Diener Zepto) for 210 s. Then a silica bead was glued to the end of the cantilever with UHU plus endfest adhesive (UHU GmbH & Co. KG, Bühl, Germany) and a micromanipulator (Sutter Instrument Co., Novato, USA). The glue was cured for 6 h at 60 °C and the colloidal probe then sintered at 1150 °C for 3 h. The heat treatment removes organic contaminations from the glue, decreasing the bead to a size of $\approx 4.5\,\mu m$ with a significantly reduced surface roughness [22]. The complete characterization of the top and bottom beads via AFM and SEM is given in the next Section S1.2.2.

S1.2.2 Characterization of the Silica Beads

We show in this publication interaction forces measured between a single top bead and five different bottom beads. These silica beads were characterized after the sintering process and prior to the first measurement via Scanning Electron Microscopy (SEM, top bead) and Atomic Force Microscopy (AFM, bottom beads) to confirm their spherical shape and to determine the size and surface topography.

Figure S1 shows SEM images of the top bead at two different magnifications, taken with an accelerating voltage of 1.5 kV at a JSM-7600F (JEOL Ltd., Tokio, Japan). The secondary electrons were used for imaging. A circle was inscribed to the image at 20000x magnification and then the bead diameter could be estimated to 2.111 µm from the diameter of the circle and the length of the scalebar (both in pixels).

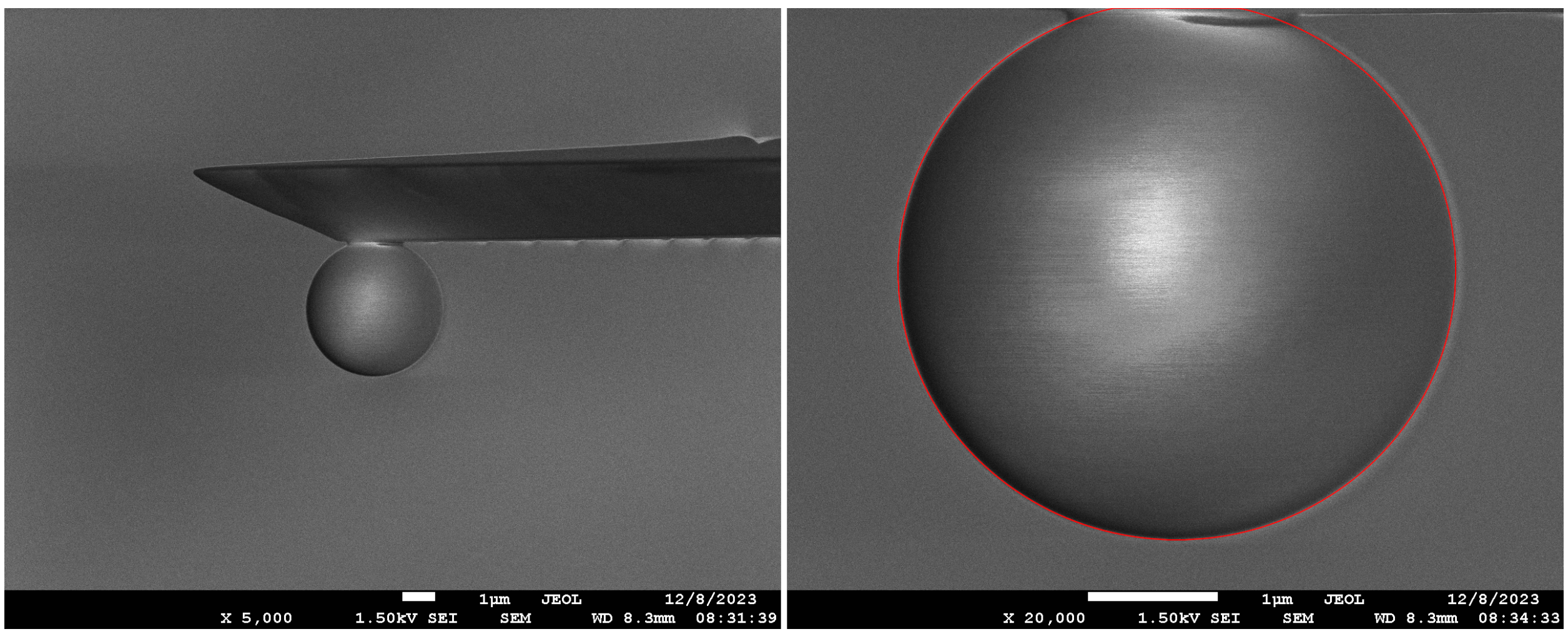

Figure S1: SEM images of the colloidal probe at 5000x (left) and 20000x (right) magnification, provided by Ulrike Kunz (Advanced Electron Microscopy Division, Technical University of Darmstadt). The radius of the inscribed circle in the right image was used together with the corresponding scalebar to determine the radius of the bead to 2.111 µm.

Figure S2 (a.1) - (e.1) shows AFM scans of the five used bottom beads, obtained in tapping mode with a free oscillation amplitude (FOA) of $\approx$ 100 nm and setpoint of 65 % of the FOA at an MFP-3D BIO AFM (Oxford Instruments Asylum Research Inc., Santa Barbara, USA) mounted on an inverted optical microscope IX71 (Evident Corporation, formerly Olympus, Tokyo, Japan). The scan size was chosen to be 1 µm with 512 points & lines and a line rate of 1 Hz. The cantilever (Olympus AC160TS-R3, $f_{\mathrm{res}} \approx 270\,\mathrm{kHz}$, $k \approx 25\,\mathrm{N\,m^{-1}}$, $r_{\mathrm{tip}} \approx 7\,\mathrm{nm}$) was driven slightly below its resonance frequency f_{res}. Each bead was scanned up to three times and the corresponding deviations are given as uncertainties in the following.

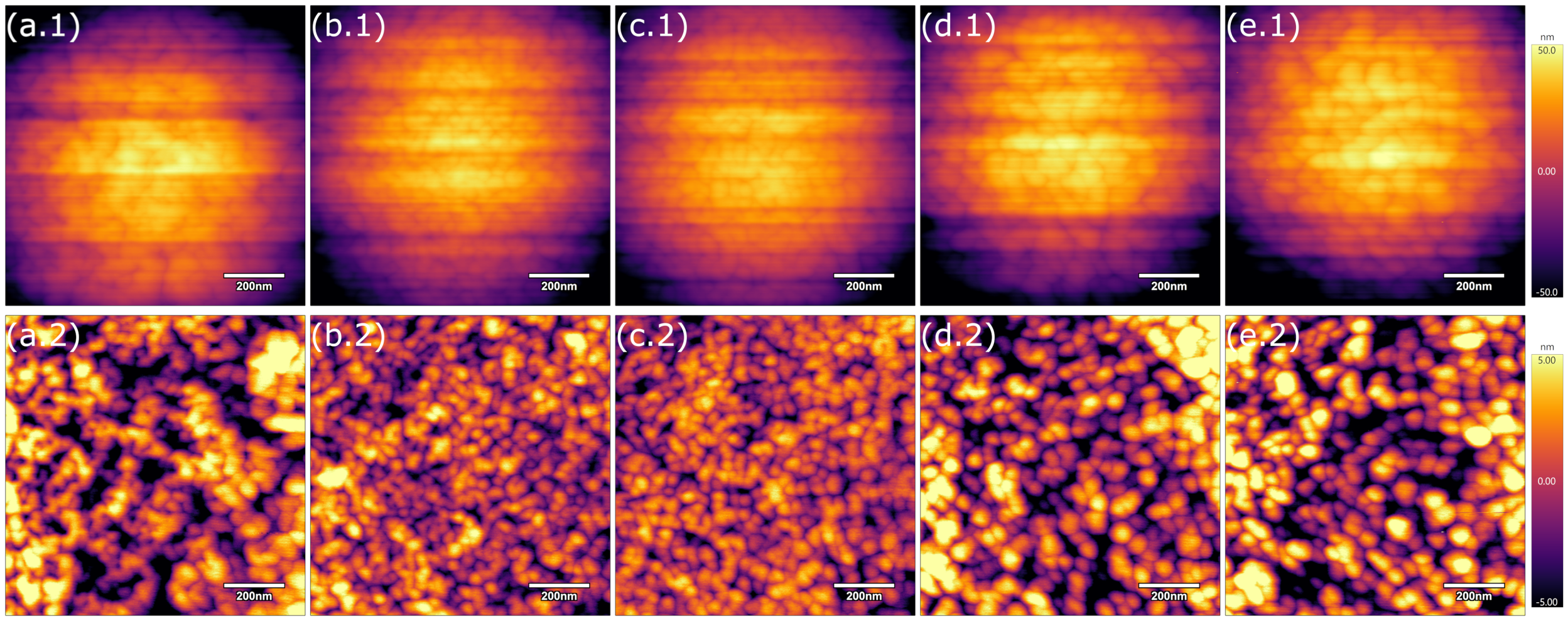

Figure S2: AFM images of the bottom beads on the quartz disk. The top row (a.1) - (e.1) shows the raw height data of the five used beads, the bottom row (a.2) - (e.2) the surface topography after subtracting a sphere cap with variable radius and a subsequent zeroth-order line flattening.

Then a sphere cap with variable center and radius was fitted to the raw height data and subtracted from it, followed by a zeroth-order line flattening. The resulting topography images are shown in Figure S2 (a.2) - (e.2) and the corresponding radii are given in Table S2.

Table S2: Radius and surface roughness of the five bottom beads as extracted from the processed AFM scans in Figure S2.

Bead	Radius µm	Surface roughness nm
(a)	2.239 ± 0.042	2.87 ± 0.02
(b)	2.277 ± 0.019	1.94 ± 0.02
(c)	2.368 ± 0.043	1.81 ± 0.01
(d)	2.162 ± 0.006	2.66 ± 0.01
(e)	2.400 ± 0.146	2.94 ± 0.13

From these topography images we then calculated the surface roughness of each bead. Therefore the rms-roughness within a 500 nm × 500 nm box with its center identical to the center of the fitted sphere cap was determined for each scan separately and the corresponding averages are given in Table S2.

S1.2.3 Colloidal Probe Atomic Force Microscopy (CP-AFM)

The interaction forces between silica beads were measured by the colloidal probe technique with a MFP-3D BIO AFM (Oxford Instruments Asylum Research Inc., Santa Barbara, USA) mounted on an inverted optical microscope IX71 (Evident Corporation, formerly Olympus, Tokyo, Japan). A sketch of the setup is depicted in Figure S3.

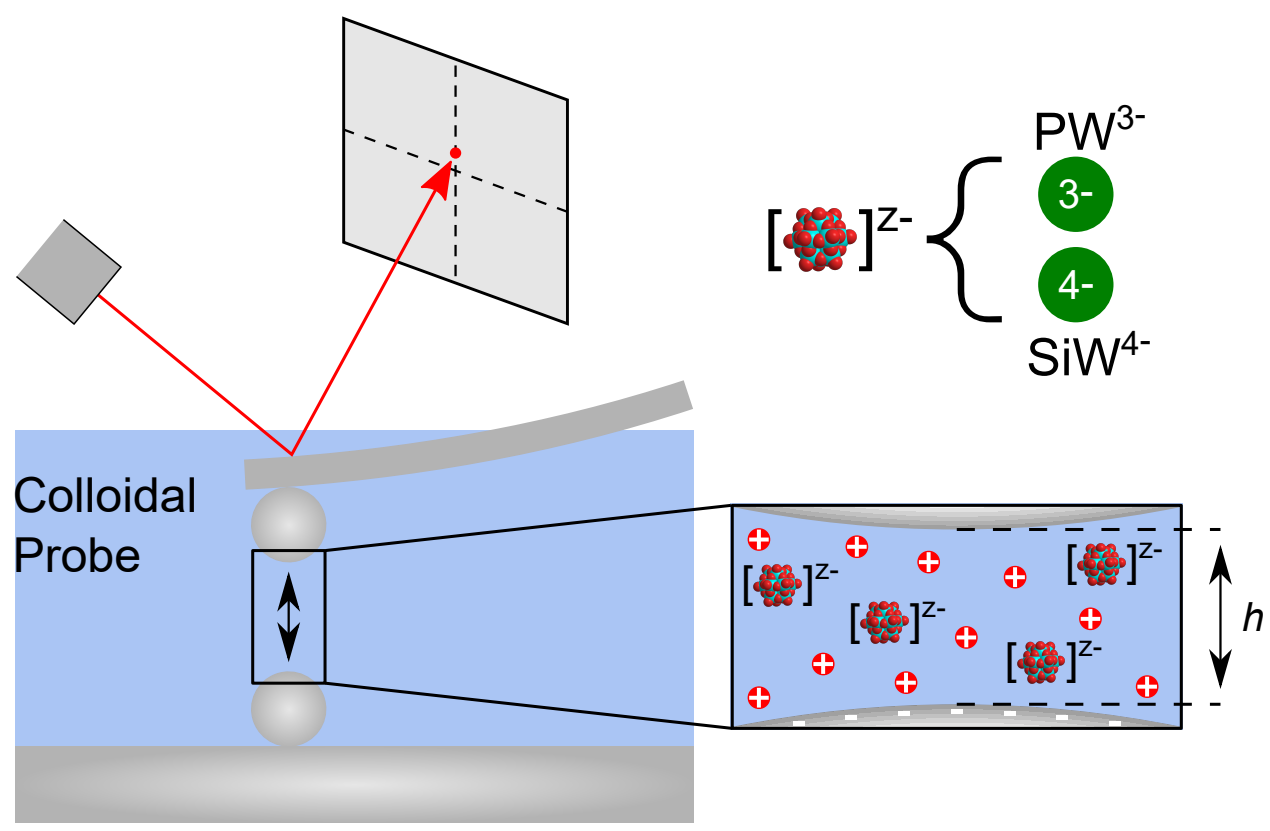

Figure S3: Schematic of the symmetric colloidal probe AFM setup used to measure the interaction forces between two silica beads. The beads at separation h confine the Keggin ions phosphotungstate $\left(PW^{3-}\right)$ and silicotungstate $\left(SiW^{4-}\right)$, the interaction force is deduced from the deflection of the cantilever. Both ions possess the same diameter of $\approx 1.1 - 1.2\,$nm [23–25], but a different charge (and therefore charge density).

The quartz disk and colloidal probe were cleaned with water and ethanol and then treated with oxygen plasma (0.4 mbar, power: 50 W, Diener Zepto) for 210 s prior to each measurement. The quartz disk was mounted in a fluid cell and the cell filled with $\approx 1\,$mL of solution. The setup was left to equilibrate at $(30 \pm 1)\,°$C for 30 min before data were collected. Then the top bead was centered over the first bottom bead via the optical microscopy image. The accuracy of the alignment was increased to $\approx 100\,$nm by performing a force map with the AFM. Then 100 force-distance curves were recorded in closed-loop mode with a travel distance of 1 µm and an approach and retraction velocity of $200\,\mathrm{nm\,s^{-1}}$ at a sample rate of 2 kHz. The force setpoint was fixed to 60 nN, corresponding to a normalized force of $\approx 55\,\mathrm{mN\,m^{-1}}$. The raw deflection vs displacement data were transformed to force vs separation curves following procedures from literature [26]. The cantilever spring constants were extracted from

the thermal noise spectrum via the Sader method [27] prior to the first experiment. The lever sensitivity was calculated from the slope of each curve above a normalized force of $10\,\mathrm{mN\,m^{-1}}$, assuming direct contact between the beads. Up to 100 force curves were stacked and then smoothed using a multipass boxcar smoother filter/function to reduce the noise and to increase the force resolution.

S1.3 Electrostatic Modeling

We describe the measured interaction forces between the charged silica beads following the DLVO theory [28–30] as a superposition of the attractive van der Waals (vdW) and the repulsive electric double layer (EDL) contribution.

$$F_{\mathrm{DLVO}} = F_{\mathrm{vdW}} + F_{\mathrm{EDL}} \tag{S1}$$

To rule out geometric influences, we make use of the Derjaguin approximation [31] and report the normalized forces.

$$\frac{F}{R_{\mathrm{eff}}}(h) = 2\pi W(h) \tag{S2}$$

h denotes the separation between the beads, $W(h)$ the interaction free energy per surface area and $R_{\mathrm{eff}} = \frac{R_1 R_2}{R_1 + R_2}$ the effective radius of the system [32, 33]. The validity of this approximation for beads in the micrometer range was demonstrated earlier [34].

The radii $R_{1,2}$ were extracted for each pair of beads separately from AFM and SEM images and are given in Table S2.

S1.3.1 Van der Waals (vdW) Force

The van der Waals (vdW) contribution was modeled using the non-retarded van der Waals force

$$\frac{F_{\mathrm{vdW}}}{R_{\mathrm{eff}}}(h) = -\frac{H}{6h^2} \tag{S3}$$

with Hamaker constant H [35]. The Hamaker constant was determined from measurements in aqueous 500 mM NaCl solutions prior to the actual experiments for each combination of beads separately, giving values in the range of approximately $(0.6 - 1.9) \times 10^{-21}$ J. This aligns well with results from literature [22, 36]. The exact value for each combination of beads can be found in Table S3.

S1.3.2 Electric Double Layer (EDL) Force

The second contribution arises from the overlapping electric double layers (EDLs) of both silica surfaces. We consider the symmetric case of two parallel, equally charged plates at separation h, immersed in an electrolyte solution containing POMs and the salt NaCl. The charged surfaces create an electric field within the gap, prompting the ions in the diffuse layer to arrange according to a Boltzmann distribution. A schematic depiction of that situation is given in Figure S4.

The mathematical description of the diffuse layer is provided by the Poisson-Boltzmann (PB) equation [32, 33, 38]

$$\frac{\mathrm{d}^2\psi}{\mathrm{d}x^2} = -\frac{e}{\varepsilon\varepsilon_0}\sum_i z_i c_{i,0} \exp\left(\frac{-e z_i \psi}{k_\mathrm{B}T}\right) \tag{S4}$$

with the elementary charge e, relative permittivity of water ε ($\varepsilon = 76.66$ at 30 °C, averaged from [39–41])), vacuum permittivity ε_0, Boltzmann constant k_B and absolute temperature T. The sum runs over all ionic species present in the electrolyte with valences z_i and bulk concentrations $c_{i,0}$. x is the spatial coordinate between the two plates located at $x = \pm\frac{h}{2}$. The solution for ψ was determined numerically using a script provided by Dr. Plinio Maroni, University of Geneva, which was already successfully applied for similar experiments [42, 43]. To account for charge regulation effects, we applied the constant regulation approximation

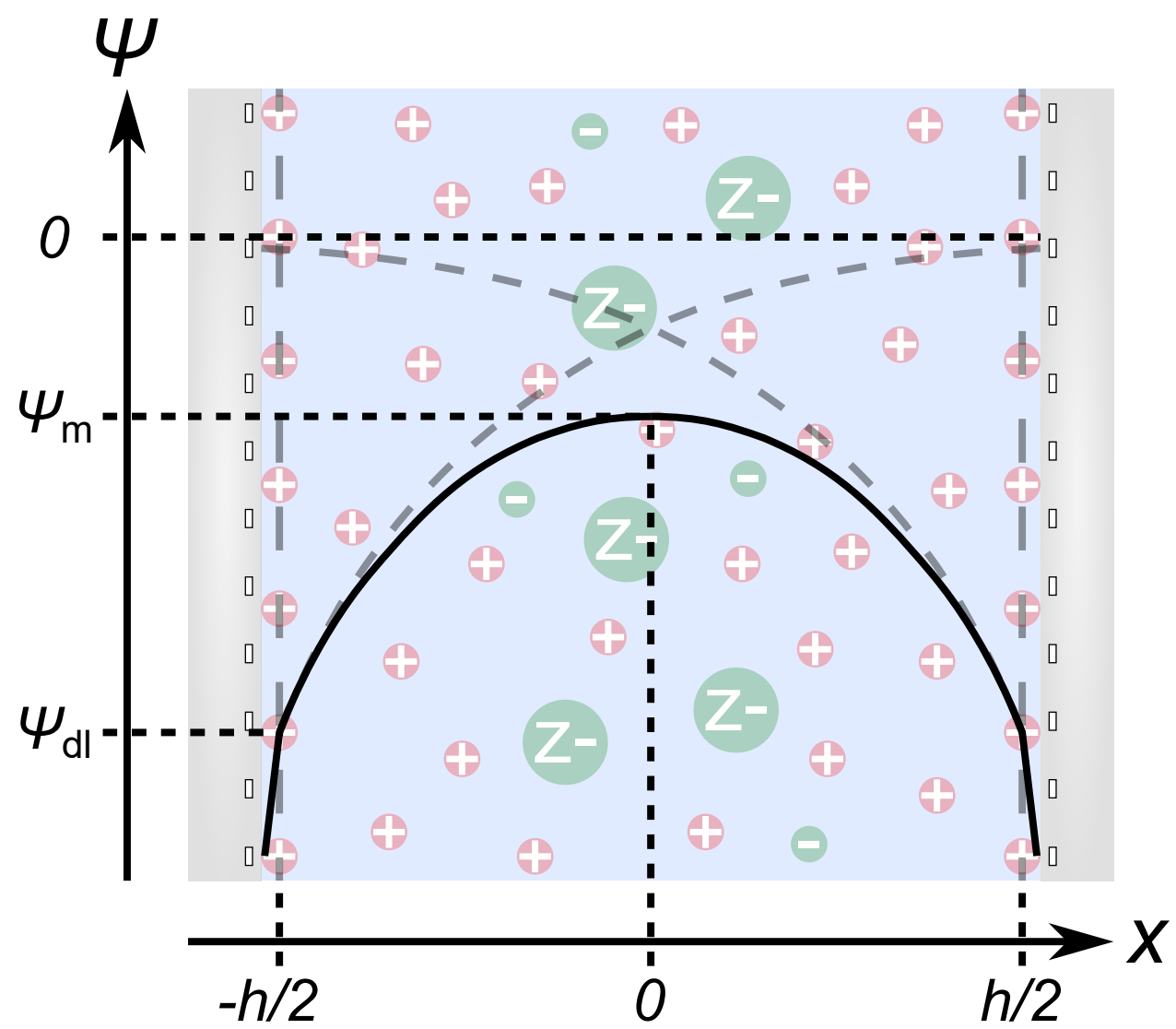

Figure S4: Schematic representation of the basic Stern model [37] for the EDL between two negatively charged silica surfaces immersed in solutions containing POMs and the salt NaCl. Ions directly adsorbed in the *Stern layer* partially neutralize the surface charge, while the remaining charge is compensated by free ions within the adjacent *diffuse layer*. The electric potential (solid black curve) decreases linearly across the Stern layer, reaching the diffuse layer potential ψ_{dl} at the origin of the diffuse layer. It then decays nonlinearly within the diffuse layer toward the midplane potential ψ_{m}, governed by the PB Equation (S4). The grey dashed curves represent the hypothetical electric potentials of the two isolated silica surfaces.

for the fitting procedure [44, 45]. This approximation introduces a fit parameter

$$p = \frac{C_{\mathrm{dl}}}{C_{\mathrm{dl}} + C_{\mathrm{in}}} \in [0, 1] \,, \tag{S5}$$

relating the capacitances of inner (C_{in}) and diffuse layer (C_{dl}). The limiting case $p = 0$ reproduces constant potential (CP) and $p = 1$ constant charge (CC) boundary conditions. In theory, also $p < 0$ would be possible [46], but these situations are not considered here. Further fit parameters are the electric potential at the origin of the diffuse layer ψ_{dl} and the ionic strength of the solution I_{fit}. The latter one can then be compared to its nominal value defined as

$$I_{\mathrm{nom}} = \frac{1}{2} \sum_i c_{i,0} z_i^2 = I_{\mathrm{nom}}^{\mathrm{POM}} + I_{\mathrm{nom}}^{\mathrm{NaCl}} \,, \tag{S6}$$

assuming that the contributions from the POMs and the added salt are additive. The ionic

strength provides access to the inverse Debye length [32, 33, 47]

$$\kappa^2 = \frac{2e^2 I_{\mathrm{fit}}}{\varepsilon\varepsilon_0 k_{\mathrm{B}} T}, \tag{S7}$$

a measure for the range of the linearized long-range decay of the potential. From charge neutrality we can deduce that the charge density σ_{dl} and potential ψ_{dl} of the diffuse layer are related via the Grahame equation [48]

$$\sigma_{\mathrm{dl}} = \pm \left[2k_{\mathrm{B}} T \varepsilon\varepsilon_0 \sum_i c_{i,0} \left(\exp\left\{ -\frac{z_i e \psi_{\mathrm{dl}}}{k_{\mathrm{B}} T} \right\} - 1 \right) \right]^{\frac{1}{2}}. \tag{S8}$$

Once ψ is known, the electric double layer force follows by integration of the disjoining pressure Π_{EDL} and Derjaguin's approximation [43, 49]

$$\frac{F_{\mathrm{EDL}}}{R_{\mathrm{eff}}}(h) = -2\pi \int_{\infty}^{h} \Pi_{\mathrm{EDL}}(h')\, \mathrm{d}h'. \tag{S9}$$

S2 Determination of the Hamaker Constant

The magnitude of the van der Waals (vdW) force is not only dependent on the material of the beads (amorphous silica), also other parameters such as their morphology and surface roughness have a significant influence [22]. Therefore we measured the vdW force for each pair of beads separately in a 500 mM NaCl solution. In this concentration regime, electrostatic forces are completely screened, leaving the vdW contribution behind. The choice of salt does not have an influence on the interaction [36].

Figure S5 shows an exemplary force curve for one of the bottom beads in linear and double-logarithmic scale. The black solid line shows a fit according to the non-retarded vdW force from Equation (S3), proving a good agreement between data and fit model down to < 2 nm.

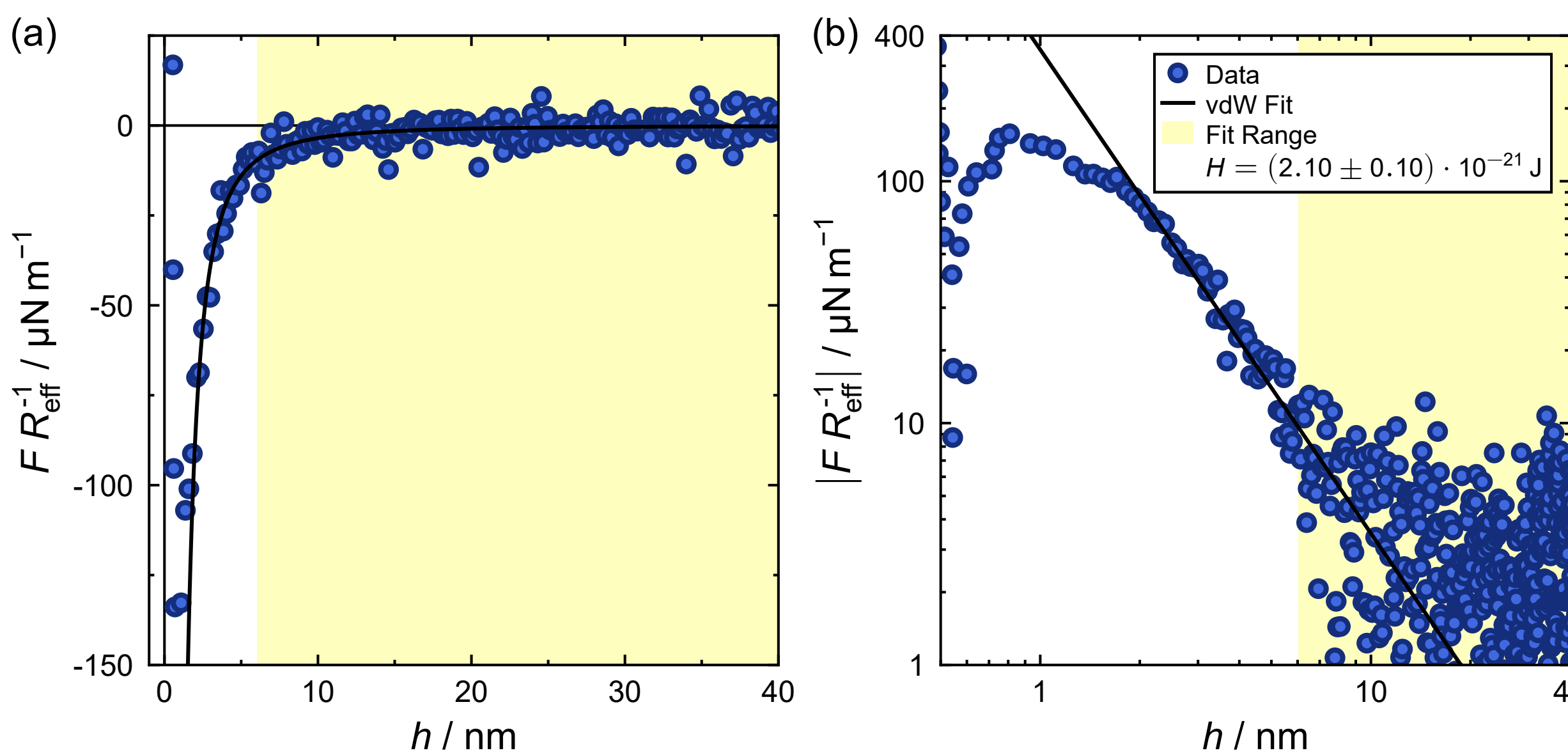

Figure S5: Interaction forces between the top bead and an exemplary bottom bead from Figure S2 in 500 mM NaCl solution. The van der Waals force was fitted according to Equation (S3) to extract the Hamaker constant H.

The extracted Hamaker constants H are listed in Table S3. They are all of a similar order of magnitude ($\approx 10^{-21}$ J) and compare well to results from literature obtained using identical types of silica beads [22, 36]. The deviations from bead to bead are expected to

arise from slight inhomogeneities in material and surface roughness of the single beads.

Table S3: Averaged Hamaker constant H for each combination of top and bottom bead as extracted from van der Waals fits to two independent measurements.

Bottom bead	Hamaker constant H in 10^{-21} J
(a)	1.25 ± 0.03
(b)	1.90 ± 0.07
(c)	1.52 ± 0.05
(d)	1.38 ± 0.04
(e)	0.58 ± 0.03

S3 Polyoxometalates and NaCl at 100 mM

To demonstrate that our colloidal probe setup can resolve screening lengths of ≈ 1 nm, as encountered in electrolyte solutions with a nominal ionic strength of 100 mM, we compare the interactions measured for both polyoxometalates (POMs) with those for NaCl. Figure S6 displays a clear difference between the force curves in POM solutions and those in the simple electrolyte, despite I_{nom} being the same. The interaction is purely attractive for PW^{3-} and SiW^{4-} down to separations below 1 nm, dominated by the van der Waals (vdW) attraction. In contrast, NaCl clearly shows a residual electric double layer force, in line with similar observations from literature for 100 mM ionic strength [50–52].

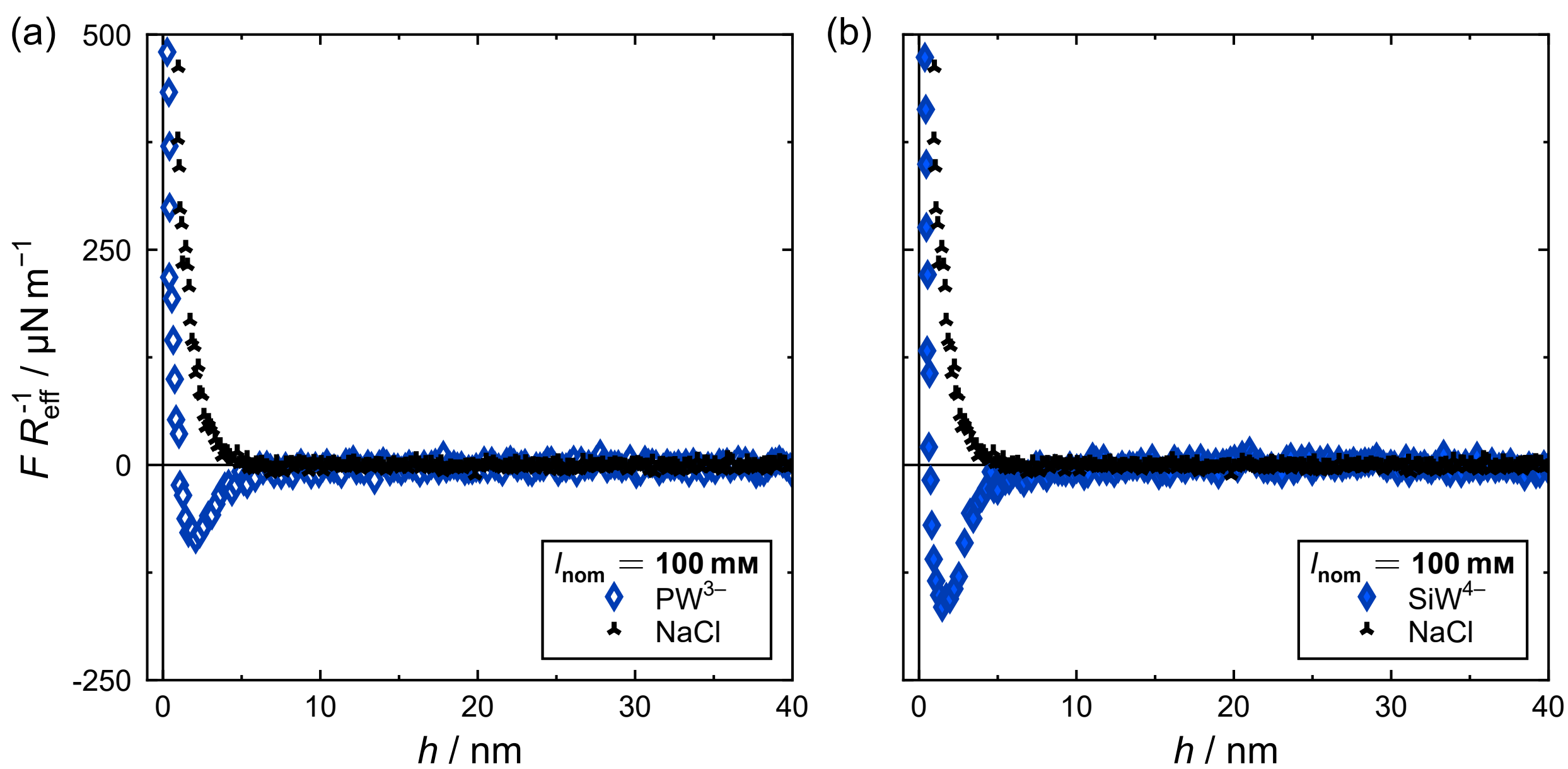

Figure S6: Interaction forces between silica beads in aqueous solutions of the two Keggin ions (a) PW^{3-} and (b) SiW^{4-} at I_{nom} = 100 mM, compared to NaCl at the same ionic strength. The data for the POMs is taken from Figure 3. While NaCl exhibits a residual electric double layer repulsion, this repulsion is absent for the POM solutions.

The lack of observable electric double layer repulsion in the POM solutions indicates that the electrostatic screening is stronger than anticipated, rather than reflecting an artifact of limited experimental resolution.

S4 Surface Charge Density

From the fit parameters depicted in Figures 2 and 4 (c) - (e), we are able to calculate the charge density σ_{dl} at the origin of the diffuse layer (herein denoted as surface charge density) according to the Grahame Equation (S8). This surface charge density comprises the bare silica charge plus the charge of the ions directly adsorbed within the Stern layer (see Figure S4) and is heavily pH-dependent. Therefore, we begin our consideration by determining the pH values of all polyoxometalate (POM) solutions before we continue with a discussion of sign and absolute value of the surface charge density.

S4.1 pH Measurements

We measured the pH of all pure PW^{3-} and SiW^{4-} solutions using an inoLab pH 720 pH meter and a SenTix 42 pH electrode (both: Xylem Analytics Germany Sales GmbH & Co. KG, Weilheim, Germany) at 23.5 °C. The results are given in Table S4 and Figure S7.

Table S4: pH of the investigated pure POM solutions.

Electrolyte	Ionic strength I_{nom} mM	pH
PW^{3-}	0.5	3.33
	2.5	2.71
	5	2.40
	10	1.99
	25	1.69
	50	1.47
	100	1.04
SiW^{4-}	0.5	2.98
	2.5	2.40
	5	2.19
	10	1.97
	25	1.63
	50	1.43
	100	1.22

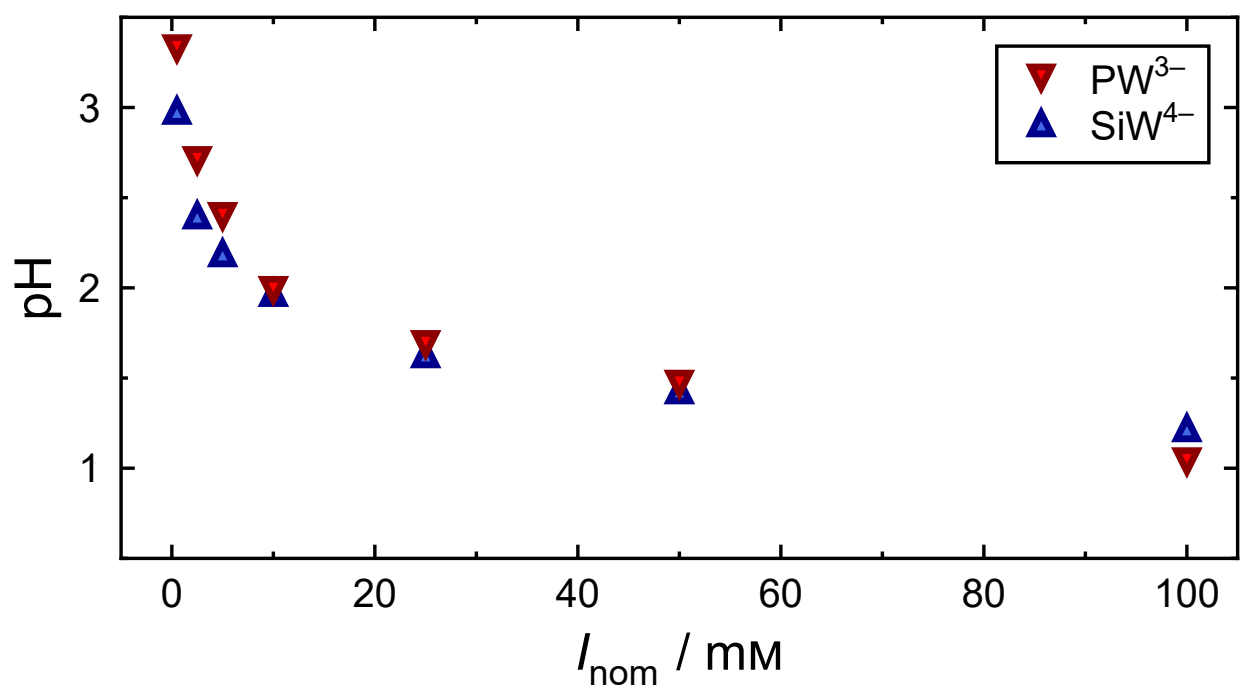

Figure S7: pH of the investigated pure POM solutions as given in Table S4.

These values are in agreement with results from Simons *et al.*, confirming that the H^+-counterions of the POMs are dissociated [53]. The addition of NaCl has only a minor influence on the pH of the mixed solutions.

S4.2 Sign of the Surface Charge

The employed symmetric model does not allow the unambiguous determination of the sign of the surface charge. This choice has to be made according to known properties of the specific solid-liquid interface. The molecular structure of silica leads to two different chemical groups being exposed to the surrounding liquid, Si_2O and SiOH (silanol) [54–56]. In the pH range investigated here (pH > 0), the Si_2O groups can be considered as inert against protonation and do not contribute to the surface charge [57, 58]. In turn, the silanol groups are present as two different types, differing in their orientation relative to the interface (in- and out-of-plane) as well as their ability to form hydrogen bonds and therefore their acidity [59, 60]. Depending on the pH history of the silica, even a third species of silanol groups is reported [61]. For decreasing pH value, all types of silanol groups undergo the same two-step protonation reaction

$$\mathrm{SiO^- + H^+ \rightleftharpoons SiOH} \tag{S10a}$$

$$\mathrm{SiOH + H^+ \rightleftharpoons SiOH_2^+} \tag{S10b}$$

from a negative charge, over being neutral to a positive charge [55, 58, 62–64]. The exact pK_a values of these protonation reactions and the surface charge density vary depending on the concentration and type of electrolyte [65–70]. It has also been demonstrated that the exact value of the isoelectric point (IEP) can depend on the specific device which is used to measure it [71, 72]. However, most experimental and theoretical studies have either revealed that the points of zero charge (PZCs) or IEPs of quartz and silica lie between $1-3$ or at least excluded that they can be found at pH > 3 [60, 63, 73–79].

To elucidate the pH dependence of the surface charge in our system, we performed reference measurements in pH-adjusted KCl solutions at a constant ionic strength of $I_{\text{nom}} =$ 10 mM. The solution composition was varied gradually from nearly pure KCl at pH $= 5$ to pure HCl at pH $= 2$, using appropriate KCl/HCl mixtures for intermediate pH values. The resulting interaction forces are depicted in Figure S8 (a) and exhibit electrostatic double layer repulsion throughout the entire investigated pH range. The similar slopes of all force curves are consistent with the constant ionic strength of the solutions. In contrast, the magnitude of the repulsion decreases monotonically with decreasing pH. Lowering the pH provides no indication of a reversal of the surface charge as described by Equation (S10b). This would lead to a characteristic reentrant behavior, where the electrostatic repulsion vanishes at a certain HCl concentration and appears again at higher concentrations [80–85]. Therefore, the surface potential remains negative throughout the whole measurement series down to at least pH $= 2$. This conclusion is further supported by results from Guleryuz *et al.* [86], who demonstrated an electrostatic repulsion between colloidal silica beads down to at least pH $= 2.7$ and Ducker *et al.* [51], proving a negative surface charge for silica down to even pH $= 2$. Moreover, the remaining repulsion at pH $= 2$ in Figure S8 (a) is still substantial, suggesting that the negative surface charge likely persists down to pH $= 1$, the lowest pH encountered in the POM solutions (*c.f.* Section S4.1).

To quantify the aforementioned trends, the data were fitted numerically using DLVO theory. Figure S8 (b) shows the extracted diffuse layer potential ψ_{dl} as well as the surface

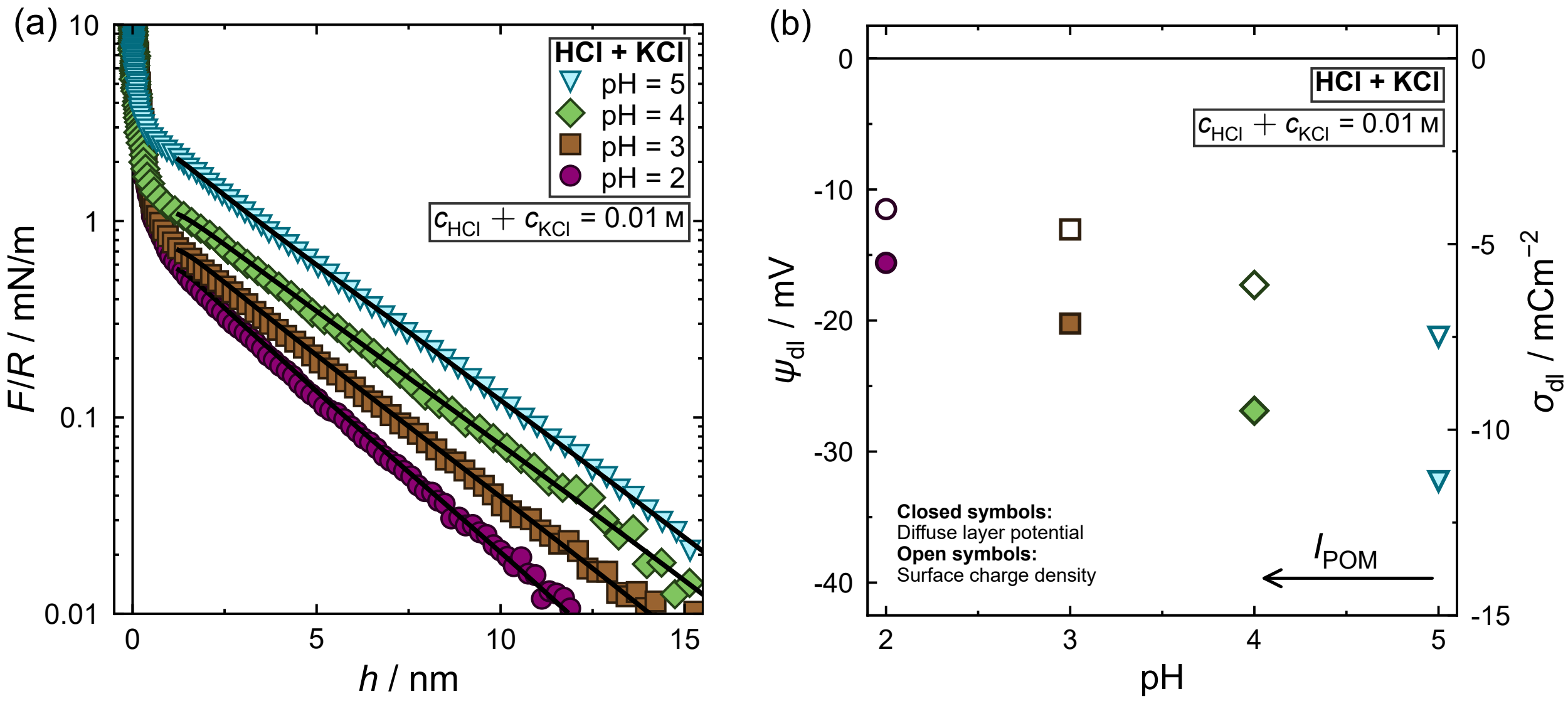

Figure S8: Reference measurements in pH-adjusted KCl solutions: (a) Interaction forces between the silica beads and (b) the corresponding diffuse layer potentials ψ_{dl} as well as surface charge densities σ_{dl}. The pH of the aqueous solutions was adjusted by the addition of HCl while keeping the total ion concentration $c_{tot} = c_{HCl} + c_{KCl} = 10\,\text{mM}$ constant. The closed symbols in (a) correspond to data points, the black lines to numerical fits according to the DLVO theory (Equation (2)). Closed symbols in (b) denote ψ_{dl} and open symbols σ_{dl}. Please note for comparison with the POM data that an increase in POM concentration decreases the pH value (from right to left).

charge density σ_{dl} calculated according to Equation (S8). As discussed before, the negative sign in Equation (S8) can be assumed. Both the absolute values of ψ_{dl} and σ_{dl} in HCl/KCl solutions decrease monotonically with pH, in accordance with Equation (S10a). The trend in ψ_{dl} is similar to the one observed for the POM solutions, *c.f.* Figure 2 (c).

The POM solutions with the lowest ionic strength (and therefore concentration of the Keggin ions) exhibit the highest pH, corresponding to pH = 3.3 for PW^{3-} and pH = 3.0 for SiW^{4-} (*c.f.* Section S4.1). Figure 2 (a) & (b) shows a strong electrostatic repulsion for both systems at this ionic strength and pH, providing evidence for the presence of surface charges on the silica beads. These charges must be negative. As for HCl/KCl, increasing the ionic strength of the solutions gives no hints for a reversal of the surface charge. Therefore, the surface charge remains negative throughout the whole measurement series and a negative sign in Equation (S8) can be assumed. Similar arguments hold for the mixed SiW^{4-} and NaCl

solutions. The surface charge of silica is negative for the pristine SiW^{4-} solutions, as discussed before. The data in Figure 4 (a) & (b) show a clear electrostatic repulsion throughout all of the studied systems, without any hint for a reentrant behavior indicating a charge reversal.

S4.3 Pure Polyoxometalates

The surface charge density σ_{dl} for the pure POM solutions is shown in Figure S9 (a). The absolute value $|\sigma_{dl}|$ is of the same order of magnitude and exhibits a similar trend for both systems, PW^{3-} and SiW^{4-}. With increasing nominal ionic strength of the solution, $|\sigma_{dl}|$ increases in magnitude. PW^{3-} shows an initial increase with a maximum around $I_{nom}\left(PW^{3-}\right) = 2.5\,\text{mM}$, a drop at $10\,\text{mM}$ and then $|\sigma_{dl}|$ continues to increase. The development for SiW^{4-} doesn't show the intermediate drop, rather $|\sigma_{dl}|$ is monotonically increasing.

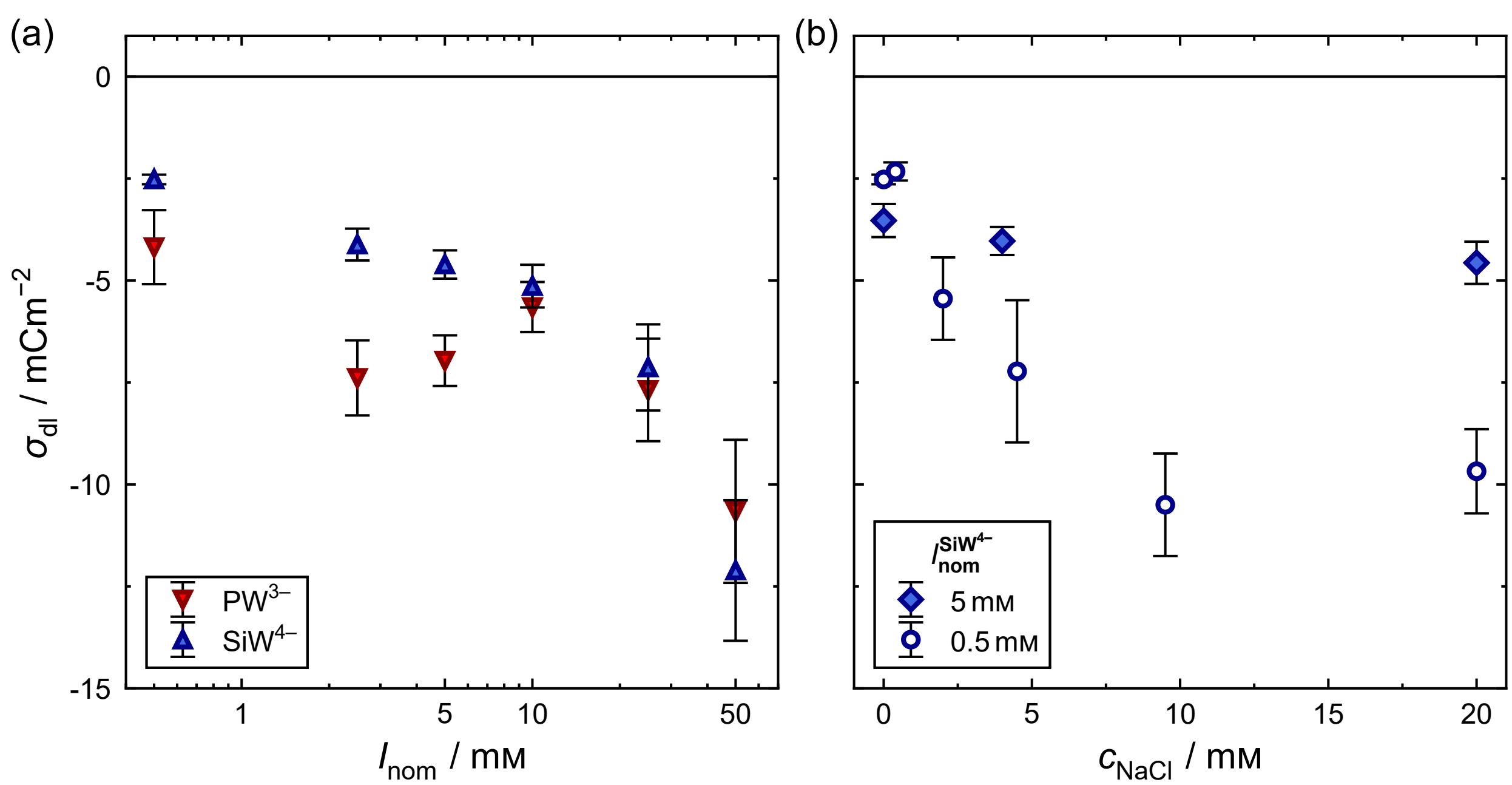

Figure S9: Charge density σ_{dl} of the two silica beads at the origin of the diffuse layer for (a) the two types of Keggin ions and (b) the two lowest ionic strengths of SiW^{4-} in dependence of the concentration of added NaCl. Calculated from the fit parameters from Figures 2 and 4 (c) - (e), respectively, according to Equation (S8).

The charge density of silica in aqueous electrolytes is known to depend on the choice of the electrolyte as well as its ionic strength [66–68]. Typically, an increase in ionic strength

of the solution gives rise to an increase in charge density [65, 66, 71, 72, 87]. This can be explained by the reduced repulsion between the surface charges due to the improved electrostatic screening, reducing the cost in free energy to create new surface charges [87–89]. Furthermore, for the studied POMs the increase in ionic strength is accompanied by a decrease in pH due to the increased number of protons as counterions, *c. f.* Section S4.1. As previously discussed, the protonation-deprotonation equilibrium of the silanol groups at the silica surface is highly pH-dependent and provides a second pathway to modify the surface charge density. A decrease in pH from neutral or slightly acidic conditions to more acidic conditions typically leads to a decrease in $|\sigma_{\mathrm{dl}}|$ [61, 65–68, 71, 72, 87], in agreement with our observations from the last Section S4.2 (*c. f.* Figure S8 (b)). A third pathway specifically available for Keggin ions is the direct adsorption to hydrophilic surfaces as demonstrated for mica [90, 91] and silica [91]. Drummond *et al.* found an irreversibly adsorbed PW^{3-} layer on silica [91]. Its mean thickness saturated in the mM range already at one molecular diameter, so for the solutions with an ionic strength above $\approx 2.5 - 5$ mM we expect an adsorption of the Keggin ions to the silica beads.

These three effects will compete. Due to the opposing trends for HCl ($|\sigma_{\mathrm{dl}}|$ decreases with decreasing pH, *c. f.* Figure S8 (b)) and the POMs ($|\sigma_{\mathrm{dl}}|$ increases with decreasing pH, *c. f.* Figure S9 (a)), we hypothesize that the reduced cost in free energy to create new surface charges and direct Keggin ion adsorption overcompensate the classical pH-induced reduction in silica surface charge. This is possible, since the negative charge of the silica particles becomes small at low pH and the superchaotropic anions have a strong tendency to adsorb even at likely charged interfaces. This phenomenon is known from, *e. g.*, classical chaotropic anions which adsorb at negatively charged fluid interfaces [92, 93].

S4.4 Polyoxometalates with Salt

The surface charge density σ_{dl} for mixtures of SiW^{4-} and NaCl is shown in Figure S9 (b). For both concentrations of SiW^{4-}, $|\sigma_{\mathrm{dl}}|$ increases with the NaCl concentration. Adding NaCl

increases the overall ionic strength of the solution, thus the increase in $|\sigma_{\mathrm{dl}}|$ is in agreement with the observations for the pure POM solutions from the previous Section S4.3 and also literature [65, 66, 71, 72, 87]. However, the influence of NaCl on $|\sigma_{\mathrm{dl}}|$ is more pronounced for 0.5 mM of SiW^{4-} than for 5 mM. This is likely due to the fact that the relative contribution of NaCl to the total ionic strength of the solution is larger in the first case. For pure POM solutions, $|\sigma_{\mathrm{dl}}|$ increases with SiW^{4-} content. Figure S9 (b) demonstrates that this reverses upon the addition of salt. For $c\,(\mathrm{NaCl}) \geq 2\,\mathrm{mM}$, the absolute value of the charge density for $I_{\mathrm{nom}}\left(\mathrm{SiW}^{4-}\right) = 0.5\,\mathrm{mM}$ has increased to $> 5\,\mathrm{mC\,m^{-2}}$, a value never reached for 5 mM of SiW^{4-}. This contradicts the general trend of an increase in charge density for an increase in ionic strength.

S5 Fit Parameters: Alternative Representations

S5.1 Pure Polyoxometalates

To facilitate direct comparison between the two polyoxometalates (POMs), we consistently report results in terms of the nominal ionic strength I_{nom} throughout the main text. For readers who may find it more intuitive to consider the POM concentration c_{POM} instead, the corresponding fit parameters for the pure POM solutions are provided in Figure S10 as a function of concentration.

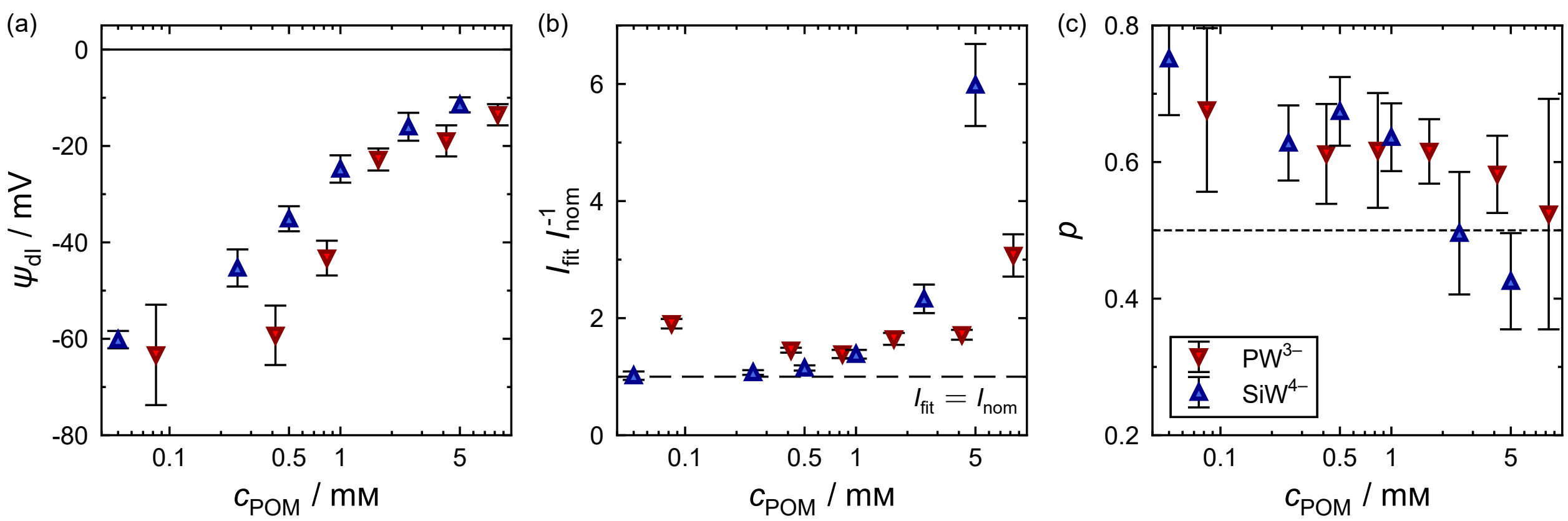

Figure S10: Fit parameters of the EDL force for the data shown in Figure 2 (a) & (b): (a) diffuse layer potential ψ_{dl}, (b) normalized ionic strength $I_{\text{fit}} I_{\text{nom}}^{-1}$ and (c) regulation parameter p in dependence on the POM concentration c_{POM}. The dashed line in (b) corresponds to the fitted ionic strength being equal to the nominal one, in (c) to $p = 0.5$. This figure is an alternative representation of Figure 2 (c) - (e).

S5.2 Polyoxometalates with Salt

For readers who may find it more intuitive to consider the concentration of added salt c_{NaCl} instead of I_{nom}, the corresponding fit parameters for the POM solutions with added salt are provided in Figure S11 as a function of salt concentration.

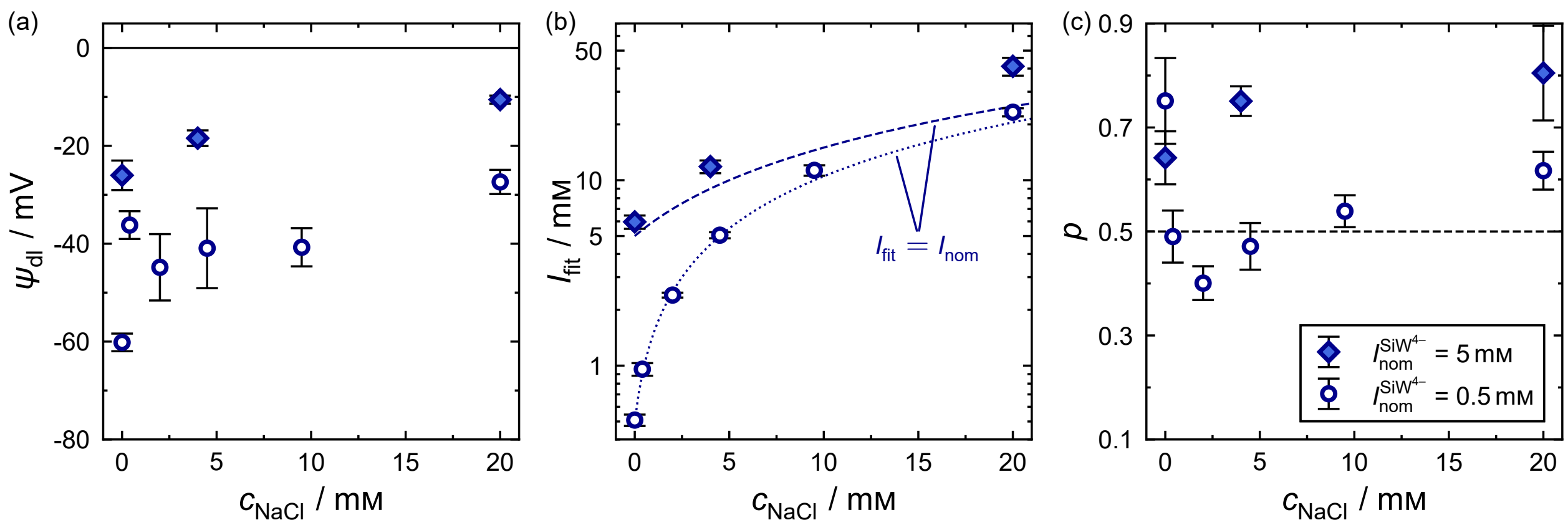

Figure S11: Fit parameters of the EDL force for the data from Figure 4 (a) & (b). The solutions contain 0.5 mM and 5 mM SiW^{4-}, respectively, and the salt NaCl. The three plots show (a) the diffuse layer potential ψ_{dl}, (b) the fitted ionic strength $I_{fit}^{SiW^{4-}}$ and (c) the regulation parameter p for two different nominal ionic strengths of SiW^{4-}. The colored dashed lines in (b) show the two nominal ionic strengths of SiW^{4-}, the black one in (c) $p = 0.5$. This figure is an alternative representation of Figure 4 (c) - (e).

S6 Electrical Conductivity Measurements

We measured the electrical conductivity of all solutions using a inoLab Cond 720 conductivity meter and a TetraCon 325 four-electrode-conductivity cell (both: Xylem Analytics Germany Sales GmbH & Co. KG, Weilheim, Germany) at 23.5 °C. The results for the specific conductance κ of the pure PW^{3-} and SiW^{4-} solutions are given in Table S5.

Table S5: Specific conductance κ of the investigated pure polyoxometalate (POM) solutions.

Electrolyte	Ionic strength I_{nom} mM	Specific conductance κ mS m^{-1}
PW^{3-}	0.5	17.2
	2.5	66.2
	5	115.9
	10	209.0
	25	474.0
	50	901.0
	100	1727.0
SiW^{4-}	0.5	8.0
	2.5	39.2
	5	76.9
	10	151.0
	25	365.0
	50	709.5
	100	1386.0

To account for the different concentrations of PW^{3-} and SiW^{4-} at the same I_{nom}, we used the concentrations from Table S1 and normalized the specific conductance to the molar conductivity $\Lambda_m = \frac{\kappa}{c}$. Strong electrolytes show a characteristic behavior described by Kohlrausch's law [94]

$$\Lambda_m = \Lambda_m^0 - K c^{\frac{1}{2}}. \tag{S11}$$

Figure S12 compares the molar conductivities of the POMs (a) PW^{3-} and (b) SiW^{4-}. PW^{3-} shows a nonlinear behavior over the whole concentration range, not following the expectations for a strong electrolyte. This can be attributed to its limited stability at higher pH. In contrast, SiW^{4-} follows Kohlrausch's law at low concentrations.

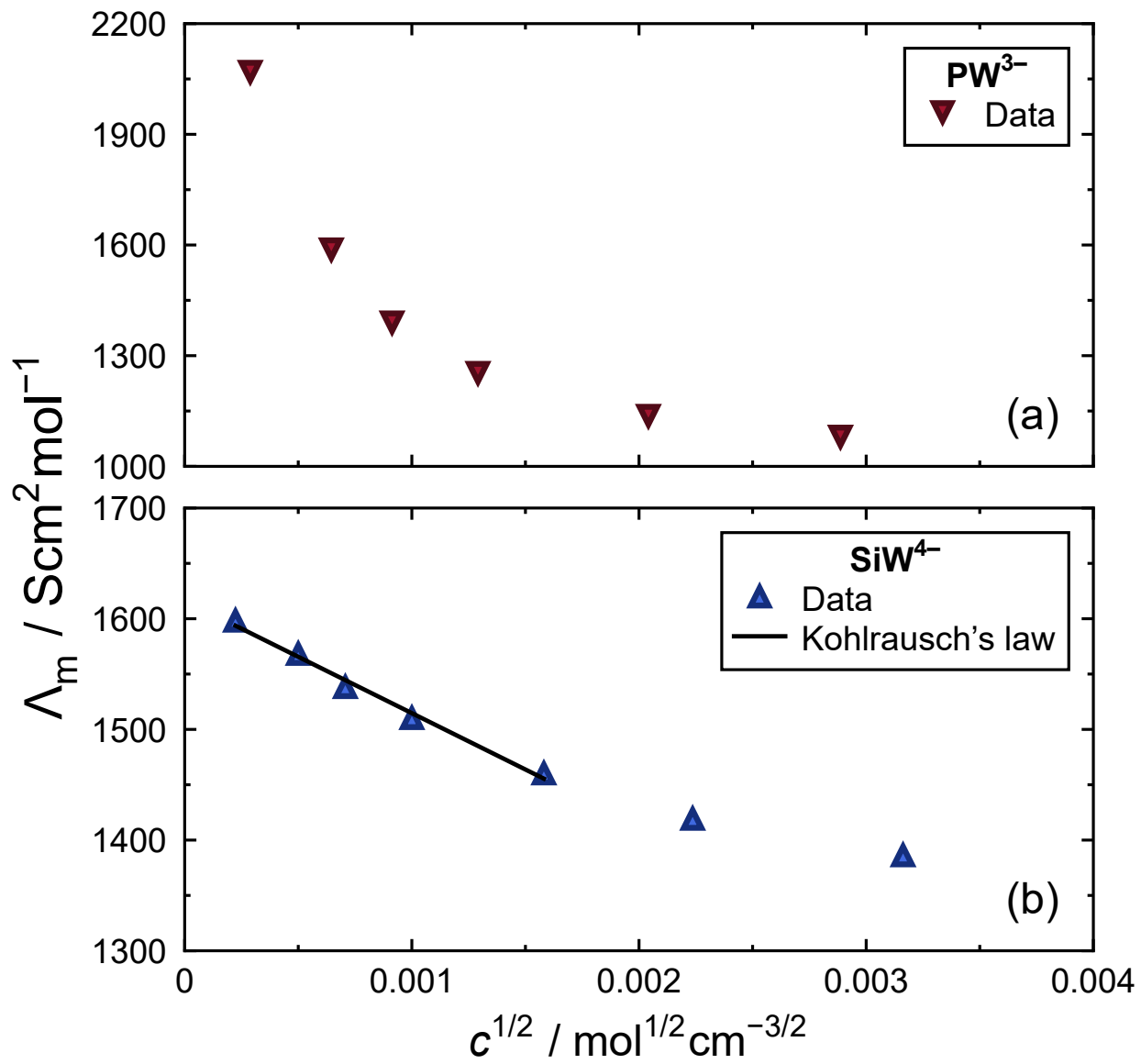

Figure S12: Molar conductivity Λ_m of aqueous solutions of the two Keggin ions (a) PW^{3-} and (b) SiW^{4-} as calculated from Table S5. The concentrations were taken from Table S1 and can be converted to I_{nom} via Equation (S6). The black line denotes a linear fit according to Kohlrausch's law (Equation (S11)).

From a linear fit to the data (black solid line) we can extract the slope and the limiting molar conductivity for SiW^{4-} as $K = -101826\,\mathrm{Scm}^{7/2}\mathrm{mol}^{-3/2}$ and $\Lambda_m^0 = 1617\,\mathrm{S\,cm^2\,mol^{-1}}$, respectively. The latter compares well to results from literature giving $\Lambda_m^0 = 1637\,\mathrm{S\,cm^2\,mol^{-1}}$ at 298 K [95].

For the sake of completeness we also determined the specific conductance κ for the mixed SiW^{4-} and NaCl solutions, which is given in Table S6 and Figure S13.

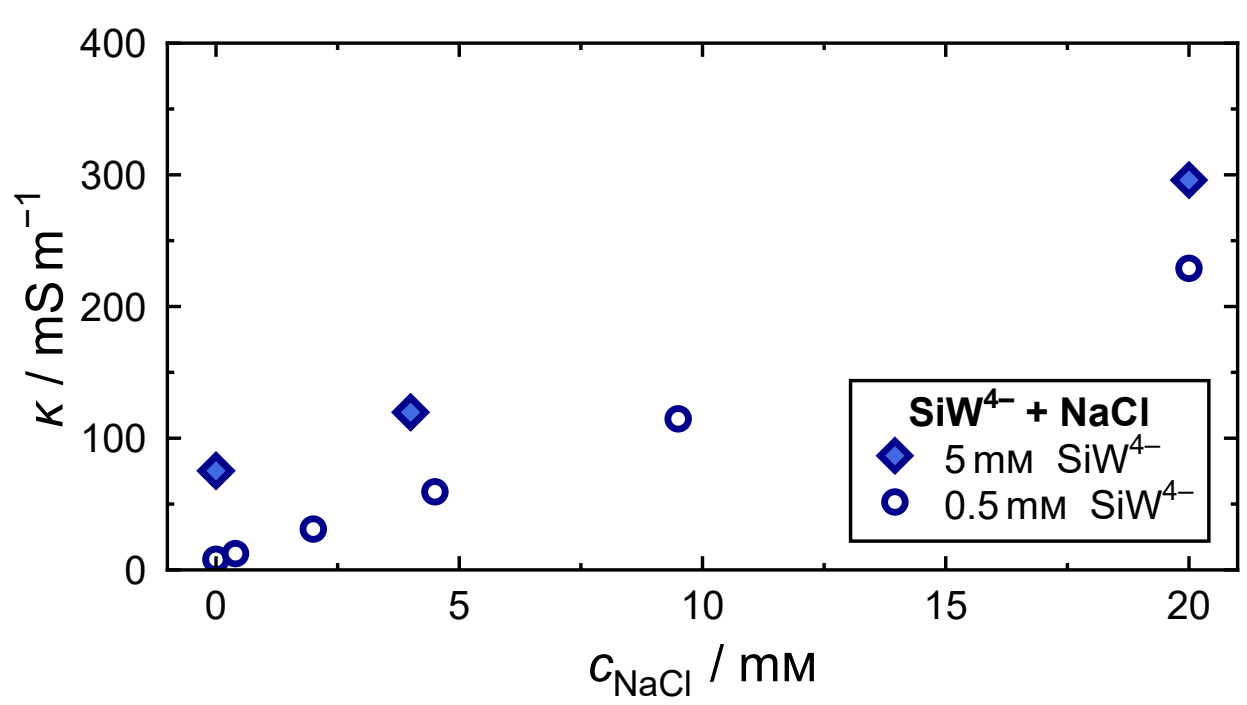

Figure S13: Specific conductance κ of the investigated mixtures of SiW^{4-} and NaCl as given in Table S6.

Table S6: Specific conductance κ of the investigated mixtures of SiW^{4-} and NaCl. A variable amount of salt was added to a fixed ionic strength of SiW^{4-}, either 0.5 mM or 5 mM.

Electrolyte	Salt concentration c_{NaCl} mM	Specific conductance κ $mS\,m^{-1}$
0.5 mM SiW^{4-} + X NaCl	0	8.0
	0.4	12.4
	2	31.0
	4.5	59.3
	9.5	114.7
	20	229.0
5 mM SiW^{4-} + X NaCl	0	75.2
	4	119.7
	20	296.0

References

(1) Keggin, J. F. The structure and formula of 12-phosphotungstic acid. *Proc. R. Soc. Lond. A* **1934**, *144*, 75–100.

(2) Misono, M.; Mizuno, N.; Katamura, K.; Kasai, A.; Konishi, Y.; Sakata, K.; Okuhara, T.; Yoneda, Y. Catalysis by Heteropoly Compounds. III. The Structure and Properties of 12-Heteropolyacids of Molybdenum and Tungsten (H3PMo12− x W x O40) and Their Salts Pertinent to Heterogeneous Catalysis. *Bull. Chem. Soc. Jpn.* **1982**, *55*, 400–406.

(3) Kanda, Y.; Lee, K. Y.; Nakata, S.-i.; Asaoka, S.; Misono, M. Solid-State NMR of H3PW12O40 · nH2O and H3PW12O40 · 6C2H5OH. *Chem. Lett.* **1988**, *17*, 139–142.

(4) Berry, F. J.; Derrick, G. R.; Mortimer, M. Identification and characterisation of stable phases of silicotungstic acid, H4SiW12O40 · nH2O. *Polyhedron* **2014**, *68*, 17–22.

(5) Olah, G. A.; Surya Prakash, G. K.; Molnár, Á.; Sommer, J. *Superacid Chemistry*; Wiley, 2009.

(6) Izumi, Y.; Matsuo, K.; Urabe, K. Efficient homogeneous acid catalysis of heteropoly acid and its characterization through ether cleavage reactions. *J. Mol. Catal.* **1983**, *18*, 299–314.

(7) Lefebvre, F.; Liu-Cai, F. X.; Auroux, A. Microcalorimetric study of the acidity of tungstic heteropolyanions. *J. Mater. Chem.* **1994**, *4*, 125.

(8) Okuhara, T.; Hu, C.; Hashimoto, M.; Misono, M. Acid Strength of Heteropolyacids and Its Correlation with Catalytic Activity. *Bull. Chem. Soc. Jpn.* **1994**, *67*, 1186–1188.

(9) Pope, M. T. *Heteropoly and Isopoly Oxometalates*; Inorganic Chemistry Concepts; Springer Berlin: Berlin, 2013; Vol. 8.

(10) Kozhevnikov, I. V.; Matveev, K. I. Heteropolyacids in Catalysis. *Russ. Chem. Rev.* **1982**, *51*, 1075–1088.

(11) Kozhevnikov, I. V. Advances in Catalysis by Heteropolyacids. *Russ. Chem. Rev.* **1987**, *56*, 811–825.

(12) Misono, M. Heterogeneous Catalysis by Heteropoly Compounds of Molybdenum and Tungsten. *Catal. Rev. Sci. Eng.* **1987**, *29*, 269–321.

(13) Okuhara, T.; Mizuno, N.; Misono, M. In *Advances in Catalysis*; Eley, D. D., Haag, W. O., Gates, B., Eds.; Academic Press, 1996; Vol. 41; pp 113–252.

(14) Jürgensen, A.; Moffat, J. B. The stability of 12-molybdosilicic, 12-tungstosilicic, 12-molybdophosphoric and 12-tungstophosphoric acids in aqueous solution at various pH. *Catal. Lett.* **1995**, *34*, 237–244.

(15) Zhu, Z.; Tain, R.; Rhodes, C. A study of the decomposition behaviour of 12-tungstophosphate heteropolyacid in solution. *Can. J. Chem.* **2003**, *81*, 1044–1050.

(16) Smith, B. J.; Patrick, V. A. Quantitative Determination of Aqueous Dodecatungstophosphoric Acid Speciation by NMR Spectroscopy. *Aust. J. Chem.* **2004**, *57*, 261–268.

(17) Maksimovskaya, R. I.; Maksimov, G. M. 31P NMR studies of hydrolytic conversions of 12-tungstophosphoric heteropolyacid. *Coord. Chem. Rev.* **2019**, *385*, 81–99.

(18) Smith, B. J.; Patrick, V. A. Quantitative Determination of Sodium Dodecatungstosilicate Speciation by 183W Nuclear Magnetic Resonance Spectroscopy. *Aust. J. Chem.* **2002**, *55*, 281–286.

(19) Bajuk-Bogdanovic, D.; Holclajtner-Antunovic, I.; Todorovic, M.; Mioc, U. B.; Zakrzewska, J. A study of 12-tungstosilicic and 12-molybdophosphoric acids in solution. *J. Serb. Chem. Soc.* **2008**, *73*, 197–209.

(20) Bajuk-Bogdanović, D.; Uskoković-Marković, S.; Holclajtner-Antunović, I. Vibration Spectroscopy Stability Investigation of 12-Tungstosilicic Acid Solution. *J. Iran. Chem. Soc.* **2015**, *12*, 137–145.

(21) Gumerova, N. I.; Rompel, A. Polyoxometalates in solution: speciation under spotlight. *Chem. Soc. Rev.* **2020**, *49*, 7568–7601.

(22) Valmacco, V.; Elzbieciak-Wodka, M.; Besnard, C.; Maroni, P.; Trefalt, G.; Borkovec, M. Dispersion forces acting between silica particles across water: influence of nanoscale roughness. *Nanoscale Horiz.* **2016**, *1*, 325–330.

(23) Baker, M. C.; Lyons, P. A.; Singer, S. J. Velocity Ultracentrifugation and Diffusion of Silicotungstic Acid. *J. Am. Chem. Soc.* **1955**, *77*, 2011–2012.

(24) Kurucsev, T.; Sargeson, A. M.; West, B. O. Size and Hydration of Inorganic Macroions from Viscosity and Density Measurements. *J. Phys. Chem.* **1957**, *61*, 1567–1569.

(25) Pope, M. T.; Varga, G. M. Heteropoly Blues. I. Reduction Stoichiometries and Reduction Potentials of Some 12-Tungstates. *Inorg. Chem.* **1966**, *5*, 1249–1254.

(26) Senden, T. J. Force microscopy and surface interactions. *Curr. Opin. Colloid Interface Sci.* **2001**, *6*, 95–101.

(27) Sader, J. E.; Chon, J. W. M.; Mulvaney, P. Calibration of rectangular atomic force microscope cantilevers. *Rev. Sci. Instrum.* **1999**, *70*, 3967–3969.

(28) Derjaguin, B. V. A theory of interaction of particles in presence of electric double layers and the stability of lyophobe colloids and disperse systems. *Acta Phys. Chim.* **1939**, *10*, 333–346.

(29) Derjaguin, B. V., L. Landau Theory of the Stability of Strongly Charged Lyophobic Sol and of the Adhesion of Strongly Charged Particles in Solutions of Electrolytes. *Acta Phys. Chim.* **1941**, *14*, 633–662.

(30) E .J. W. Verwey; J. Th. G. Overbeek *Theory of the stability of lyophobic colloids: The interaction of particles having an electric double layer.*; Elsevier Pub. Co.: Amsterdam, 1948.

(31) Derjaguin, B. Untersuchungen über die Reibung und Adhäsion, IV. *Kolloid-Z.* **1934**, *69*, 155–164.

(32) Russel, W. B.; Saville, D. A.; Schowalter, W. R. *Colloidal dispersions*, 1st ed.; Cambridge monographs on mechanics and applied mathematics; Cambridge Univ. Press: Cambridge, 2001.

(33) Israelachvili, J. N. *Intermolecular and Surface Forces*, 3rd ed.; Elsevier Science: Burlington, 2011.

(34) Rentsch, S.; Pericet-Camara, R.; Papastavrou, G.; Borkovec, M. Probing the validity of the Derjaguin approximation for heterogeneous colloidal particles. *Phys. Chem. Chem. Phys.* **2006**, *8*, 2531–2538.

(35) Hamaker, H. C. The London—van der Waals attraction between spherical particles. *Physica* **1937**, *4*, 1058–1072.

(36) Smith, A. M.; Maroni, P.; Trefalt, G.; Borkovec, M. Unexpectedly Large Decay Lengths of Double-Layer Forces in Solutions of Symmetric, Multivalent Electrolytes. *J. Phys. Chem. B* **2019**, *123*, 1733–1740.

(37) Stern, O. Zur Theorie der elektrolytischen Doppelschicht. *Z. Elektrochem. Angew. Phys. Chem.* **1924**, *30*, 508–516.

(38) Butt, H.-J.; Kappl, M. *Surface and Interfacial Forces*, 2nd ed.; Wiley-VCH Verlag GmbH & Co. KGaA: Weinheim, 2018.

(39) Wyman, J.; Ingalls, E. N. The Dielectric Constant of Deuterium Oxide. *J. Am. Chem. Soc.* **1938**, *60*, 1182–1184.

(40) Malmberg, C. G.; Maryott, A. A. Dielectric Constant of Water from 0° to 100 °C. *J. Res. Natl. Bur. Stand.* **1956**, *56*, 1–8.

(41) Uematsu, M.; Franck, E. U. Static Dielectric Constant of Water and Steam. *J. Phys. Chem. Ref. Data* **1980**, *9*, 1291–1306.

(42) Popa, I.; Gillies, G.; Papastavrou, G.; Borkovec, M. Attractive and repulsive electrostatic forces between positively charged latex particles in the presence of anionic linear polyelectrolytes. *J. Phys. Chem. B* **2010**, *114*, 3170–3177.

(43) Montes Ruiz-Cabello, F. J.; Maroni, P.; Borkovec, M. Direct measurements of forces between different charged colloidal particles and their prediction by the theory of Derjaguin, Landau, Verwey, and Overbeek (DLVO). *J. Chem. Phys.* **2013**, *138*, 234705.

(44) Behrens, S. H.; Borkovec, M. Electrostatic Interaction of Colloidal Surfaces with Variable Charge. *J. Phys. Chem. B* **1999**, *103*, 2918–2928.

(45) Behrens, S. H.; Borkovec, M. Electric double layer interaction of ionizable surfaces: Charge regulation for arbitrary potentials. *J. Chem. Phys.* **1999**, *111*, 382–385.

(46) Borkovec, M.; Behrens, S. H. Electrostatic double layer forces in the case of extreme charge regulation. *J. Phys. Chem. B* **2008**, *112*, 10795–10799.

(47) Debye, P.; Hückel, E. Zur Theorie der Elektrolyte: I. Gefrierpunktserniedrigung und verwandte Erscheinungen. *Phys. Z.* **1923**, *24*, 185–206.

(48) Grahame, D. C. The electrical double layer and the theory of electrocapillarity. *Chem. Rev.* **1947**, *41*, 441–501.

(49) Trefalt, G.; Szilagyi, I.; Borkovec, M. Poisson-Boltzmann description of interaction forces and aggregation rates involving charged colloidal particles in asymmetric electrolytes. *J. Colloid Interface Sci.* **2013**, *406*, 111–120.

(50) Ducker, W. A.; Senden, T. J.; Pashley, R. M. Direct measurement of colloidal forces using an atomic force microscope. *Nature* **1991**, *353*, 239–241.

(51) Ducker, W. A.; Senden, T. J.; Pashley, R. M. Measurement of forces in liquids using a force microscope. *Langmuir* **1992**, *8*, 1831–1836.

(52) Smith, A. M.; Borkovec, M.; Trefalt, G. Forces between solid surfaces in aqueous electrolyte solutions. *Adv. Colloid Interface Sci.* **2020**, *275*, 102078.

(53) Simons, J.; Hazra, N.; Petrunin, A. V.; Crassous, J. J.; Richtering, W.; Hohenschutz, M. Nonionic Microgels Adapt to Ionic Guest Molecules: Superchaotropic Nanoions. *ACS Nano* **2024**, *18*, 7546–7557.

(54) Parks, G. A. In *Equilibrium concepts in natural water systems*; Stumm, W., Ed.; Advances in Chemistry Series; American Chemical Society: Washington, D.C., 1967; Vol. 67; pp 121–160.

(55) Hiemstra, T.; van Riemsdijk, W. Multiple activated complex dissolution of metal (hydr) oxides: A thermodynamic approach applied to quartz. *J. Colloid Interface Sci.* **1990**, *136*, 132–150.

(56) A. C. Lasaga In *Mineral-Water Interface Geochemistry*; Hochella, M. F., White, A. F., Eds.; Reviews in Mineralogy & Geochemistry; De Gruyter: Berlin, 1990; Vol. 23; pp 17–85.

(57) Hiemstra, T.; van Riemsdijk, W.; Bolt, G. Multisite proton adsorption modeling at the solid/solution interface of (hydr)oxides: A new approach: I. Model Description and Evaluation of Intrinsic Reaction Constants. *J. Colloid Interface Sci.* **1989**, *133*, 91–104.

(58) Hiemstra, T.; de Wit, J.; van Riemsdijk, W. Multisite proton adsorption modeling at the solid/solution interface of (hydr)oxides: A new approach: II. Application to Various Important (Hydr)oxides. *J. Colloid Interface Sci.* **1989**, *133*, 105–117.

(59) Ong, S.; Zhao, X.; Eisenthal, K. B. Polarization of water molecules at a charged interface: second harmonic studies of the silica/water interface. *Chem. Phys. Lett.* **1992**, *191*, 327–335.

(60) Sulpizi, M.; Gaigeot, M.-P.; Sprik, M. The Silica-Water Interface: How the Silanols Determine the Surface Acidity and Modulate the Water Properties. *J. Chem. Theory Comput.* **2012**, *8*, 1037–1047.

(61) Darlington, A. M.; Gibbs-Davis, J. M. Bimodal or Trimodal? The Influence of Starting pH on Site Identity and Distribution at the Low Salt Aqueous/Silica Interface. *J. Phys. Chem. C* **2015**, *119*, 16560–16567.

(62) J. A. Davis; D. B. Kent In *Mineral-Water Interface Geochemistry*; Hochella, M. F., White, A. F., Eds.; Reviews in Mineralogy & Geochemistry; De Gruyter: Berlin, 1990; Vol. 23; pp 177–260.

(63) Duval, Y.; Mielczarski, J. A.; Pokrovsky, O. S.; Mielczarski, E.; Ehrhardt, J. J. Evidence of the Existence of Three Types of Species at the Quartz−Aqueous Solution Interface at pH 0−10: XPS Surface Group Quantification and Surface Complexation Modeling. *J. Phys. Chem. B* **2002**, *106*, 2937–2945.

(64) Pokrovsky, O. S.; Golubev, S. V.; Mielczarski, J. A. Kinetic evidences of the existence of positively charged species at the quartz-aqueous solution interface. *J. Colloid Interface Sci.* **2006**, *296*, 189–194.

(65) Bolt, G. H. Determination of the Charge Density of Silica Sols. *J. Phys. Chem.* **1957**, *61*, 1166–1169.

(66) Sonnefeld, J.; Gbel, A.; Vogelsberger, W. Surface charge density on spherical silica particles in aqueous alkali chloride solutions. *Colloid Polym. Sci.* **1995**, *273*, 926–931.

(67) Dove, P. M.; Craven, C. M. Surface charge density on silica in alkali and alkaline earth chloride electrolyte solutions. *Geochim. Cosmochim. Acta* **2005**, *69*, 4963–4970.

(68) Azam, M. S.; Weeraman, C. N.; Gibbs-Davis, J. M. Specific Cation Effects on the Bimodal Acid-Base Behavior of the Silica/Water Interface. *J. Phys. Chem. Lett.* **2012**, *3*, 1269–1274.

(69) Azam, M. S.; Weeraman, C. N.; Gibbs-Davis, J. M. Halide-Induced Cooperative Acid–Base Behavior at a Negatively Charged Interface. *J. Phys. Chem. C* **2013**, *117*, 8840–8850.

(70) Azam, M. S.; Darlington, A.; Gibbs-Davis, J. M. The influence of concentration on specific ion effects at the silica/water interface. *J. Phys.: Condens. Matter* **2014**, *26*, 244107.

(71) Kosmulski, M.; Hartikainen, J.; Maczka, E.; Janusz, W.; Rosenholm, J. B. Multiinstrument study of the electrophoretic mobility of fumed silica. *Anal. Chem.* **2002**, *74*, 253–256.

(72) Kosmulski, M.; Maczka, E.; Janusz, W.; Rosenholm, J. B. Multiinstrument study of the electrophoretic mobility of quartz. *J. Colloid Interface Sci.* **2002**, *250*, 99–103.

(73) Johansen, P. G.; Buchanan, A. S. An application of the microelectrophoresis method to the study of the surface properties of insoluble oxides. *Aust. J. Chem.* **1957**, *10*, 398.

(74) Parks, G. A. The Isoelectric Points of Solid Oxides, Solid Hydroxides, and Aqueous Hydroxo Complex Systems. *Chem. Rev.* **1965**, *65*, 177–198.

(75) Li, H. C.; de Bruyn, P. L. Electrokinetic and adsorption studies on quartz. *Surf. Sci.* **1966**, *5*, 203–220.

(76) Iler, R. K. *The chemistry of silica: Solubility, polymerization, colloid and surface properties, and biochemistry*; John Wiley and Sons Inc: New York, NY, 1979.

(77) Hiemstra, T.; Venema, P.; Riemsdijk, W. H. Intrinsic Proton Affinity of Reactive Surface Groups of Metal (Hydr)oxides: The Bond Valence Principle. *J. Colloid Interface Sci.* **1996**, *184*, 680–692.

(78) Hiemstra, T. Surface complexation at mineral interfaces: Multisite and charge distribution approach. Dissertation, Wageningen University, Wageningen, 2010.

(79) Kosmulski, M. The pH dependent surface charging and points of zero charge. X. Update. *Adv. Colloid Interface Sci.* **2023**, *319*, 102973.

(80) Zohar, O.; Leizerson, I.; Sivan, U. Short range attraction between two similarly charged silica surfaces. *Phys. Rev. Lett.* **2006**, *96*, 177802.

(81) Sinha, P.; Szilagyi, I.; Montes Ruiz-Cabello, F. J.; Maroni, P.; Borkovec, M. Attractive Forces between Charged Colloidal Particles Induced by Multivalent Ions Revealed by Confronting Aggregation and Direct Force Measurements. *J. Phys. Chem. Lett.* **2013**, *4*, 648–652.

(82) Ruiz-Cabello, F. J. M.; Trefalt, G.; Maroni, P.; Borkovec, M. Accurate predictions of forces in the presence of multivalent ions by Poisson-Boltzmann theory. *Langmuir* **2014**, *30*, 4551–4555.

(83) Moazzami Gudarzi, M.; Trefalt, G.; Szilagyi, I.; Maroni, P.; Borkovec, M. Forces between Negatively Charged Interfaces in the Presence of Cationic Multivalent Oligoamines Measured with the Atomic Force Microscope. *J. Phys. Chem. C* **2015**, *119*, 15482–15490.

(84) Smith, A. M.; Maroni, P.; Borkovec, M. Attractive non-DLVO forces induced by adsorption of monovalent organic ions. *Phys. Chem. Chem. Phys.* **2017**, *20*, 158–164.

(85) Moazzami-Gudarzi, M.; Adam, P.; Smith, A. M.; Trefalt, G.; Szilágyi, I.; Maroni, P.;

Borkovec, M. Interactions between similar and dissimilar charged interfaces in the presence of multivalent anions. *Phys. Chem. Chem. Phys.* **2018**, *20*, 9436–9448.

(86) Guleryuz, H.; Røyset, A. K.; Kaus, I.; Filiàtre, C.; Einarsrud, M.-A. AFM measurements of forces between silica surfaces. *J. Sol-Gel Sci. Technol.* **2012**, *62*, 460–469.

(87) Brown, M. A.; Goel, A.; Abbas, Z. Effect of Electrolyte Concentration on the Stern Layer Thickness at a Charged Interface. *Angew. Chem. Int. Ed.* **2016**, *55*, 3790–3794.

(88) Porus, M.; Labbez, C.; Maroni, P.; Borkovec, M. Adsorption of monovalent and divalent cations on planar water-silica interfaces studied by optical reflectivity and Monte Carlo simulations. *J. Chem. Phys.* **2011**, *135*, 064701.

(89) Zhao, C.; Ebeling, D.; Siretanu, I.; van den Ende, D.; Mugele, F. Extracting local surface charges and charge regulation behavior from atomic force microscopy measurements at heterogeneous solid-electrolyte interfaces. *Nanoscale* **2015**, *7*, 16298–16311.

(90) Raj, G.; Swalus, C.; Arendt, E.; Eloy, P.; Devillers, M.; Gaigneaux, E. M. Controlling the dispersion of supported polyoxometalate heterogeneous catalysts: impact of hybridization and the role of hydrophilicity-hydrophobicity balance and supramolecularity. *Beilstein J. Nanotechnol.* **2014**, *5*, 1749–1759.

(91) Drummond, C.; Pérez-Fuentes, L.; Bastos-González, D. Can Polyoxometalates Be Considered as Superchaotropic Ions? *J. Phys. Chem. C* **2019**, *123*, 28744–28752.

(92) Schelero, N.; Hedicke, G.; Linse, P.; Klitzing, R. v. Effects of counterions and co-ions on foam films stabilized by anionic dodecyl sulfate. *J. Phys. Chem. B* **2010**, *114*, 15523–15529.

(93) Schelero, N.; von Klitzing, R. Correlation between specific ion adsorption at the air/water interface and long–range interactions in colloidal systems. *Soft Matter* **2011**, *7*, 2936.

(94) Kohlrausch, Friedrich, Maltby, Margaret Eliza *Wiss. Abh. Phys. Tech. Reichsanstalt*; Verlag von Julius Springer: Berlin, 1900; Vol. 3; pp 156–227.

(95) Horky, A.; Kherani, N. P.; Xu, G. Conductivity and Transport Properties of Aqueous Phosphotungstic and Silicotungstic Acid Electrolytes for Room-Temperature Fuel Cells. *J. Electrochem. Soc.* **2003**, *150*, A1219.
